# Evaluation of synthetic reticular hybrid meshes designed for intraperitoneal abdominal wall repair: Preclinical and *in vitro* behavior

**DOI:** 10.1371/journal.pone.0213005

**Published:** 2019-02-27

**Authors:** Verónica Gómez-Gil, Marta Rodríguez, Francisca García-Moreno Nisa, Bárbara Pérez-Köhler, Gemma Pascual

**Affiliations:** 1 Department of Surgery, Medical and Social Sciences, Faculty of Medicine and Health Sciences, University of Alcalá, Madrid, Spain; 2 Networking Biomedical Research Center on Bioengineering, Biomaterials and Nanomedicine (CIBER-BBN), Madrid, Spain; 3 Ramón y Cajal Health Research Institute (IRYCIS), Madrid, Spain; 4 Department of Medicine and Medical Specialties, Faculty of Medicine and Health Sciences, University of Alcalá, Madrid, Spain; University of Camerino, ITALY

## Abstract

**Introduction:**

Reticular hybrid meshes represent an alternative material for intraperitoneal repair of abdominal hernias. These consist of a reticular mesh coated or interwoven/knitted with inert materials. This study assesses the performance of two reticular polypropylene-containing hybrid meshes, TiMESH (coated with titanium) and DynaMesh (interwoven with polyvinylidene fluoride), *in vitro*, as well as their efficiency in adhesion prevention and tissue incorporation in an intraperitoneal model.

**Methods:**

The mesothelialization capacity of TiMESH and DynaMesh was evaluated *in vitro* and compared to that of Surgipro (reticular bare polypropylene) and Preclude (laminar expanded polytetrafluoroethylene). Mesh fragments were placed on the intact parietal peritoneum of New Zealand white rabbits (n = 24), and laparoscopy performed 7 days post-surgery. Fourteen days post-implantation, adhesions were evaluated and host tissue incorporation, macrophage response, collagen expression (immunohistochemistry/RT-PCR) and neoperitoneum formation assessed. Adhesions and omental tissue were also examined.

**Results:**

Mesh pores in reticular meshes were devoid of cells in the *in vitro* study. TiMESH, DynaMesh and Surgipro showed similar adhesion rates at 7/14 days and optimal tissue integration, with significant differences in comparison to Preclude. The greatest presence of macrophages was observed for TiMESH and was significant versus that for Preclude. Hybrid meshes revealed significantly higher collagen 1 mRNA expression in implants, with no differences in the levels of collagen 3. Omental samples from animals with a reticular mesh showed significantly greater collagen 1 mRNA levels.

**Conclusions:**

The reticular structure of a mesh limits the formation of a continuous mesothelial monolayer *in vitro*, regardless of its composition. The presence of titanium as a coating or polyvinylidene fluoride interwoven with polypropylene in a reticular structure did not prevent adhesions. The hybrid meshes showed proper integration and an increase in the mRNA Col 1 levels in the implant area compared to Surgipro or Preclude.

## Introduction

Abdominal wall hernia repair is one of the most widely performed surgical procedures [1]; it preferably involves the placement of a prosthetic material, or more traditional primary closure by open suture techniques [2].

The ideal mesh for abdominal hernia repair remains an open question. Currently, the most common material employed is reticular polypropylene (PP). Several preclinical and clinical studies have shown its satisfactory performance when placed outside the peritoneal cavity. By contrast, when implantation requires contact with visceral peritoneum, adverse effects, such as adhesion formation with possible intestinal obstruction [3]; migration to intracavitary organs [4]; or even very serious complications, such as an intestinal fistula, are likely to occur [5,6].

Microporous and non-porous laminar meshes, either synthetic or biological, offer an optimal behavior in an intraperitoneal position, with minimal adhesion formation [7]. The different behavior observed between reticular and laminar meshes is likely due to mesothelial cell colonization and deposition over the latter in contrast to the reticular pattern structure [8]. The primary limitation of these materials is their poor incorporation into host tissues.

Considering all these facts, composite materials have been designed in an attempt to develop a mesh that can perform well both intra- and extraperitoneally. These mesh materials combine two different components with specific purposes: usually one of the reticular type facing the abdominal wall to achieve adequate tissue in-growth, and the other displaying a laminar structure to guarantee appropriate behavior with respect to the peritoneal contents [9,10]. Both components are typically linked together by acrylic glue, heat-sealing or suturing.

As an alternative to these composites, new meshes defined as hybrids have been developed. These should not be confused with composites; hybrid meshes combine different components knitted or woven together to produce a single mesh structure. Of particular interest are hybrid meshes formed by the combination of polypropylene with the polymer polyvinylidene fluoride (PVDF), a highly inert polymer that shows greater long-term stability than PP [11] and a very low inflammatory response [12]. Titanium-coated polypropylene meshes are also considered hybrid meshes. In this case, the chemical element titanium is introduced as a coating of the PP filaments. Due to the great biocompatibility of this element and the reduced inflammatory reaction that it provokes [13, 14], titanium appears to be a good option for use as part of a mesh capable of being implanted in different locations.

Despite the reticular pattern of these hybrid meshes, they have been specifically designed to be placed inside the peritoneal cavity and thus in contact with the visceral peritoneum. However, both preclinical and clinical studies have reported controversial results regarding their behavior, in particular with respect to adhesion formation [15–19].

Therefore, the aim of the present experimental research study was to assess the *in vitro* and *in vivo* performance of two hybrid meshes developed for intraperitoneal use: DynaMesh (PP + PVDF) and TiMESH (PP + titanium). As controls, we included an untreated traditional reticular PP mesh as well as a non-porous laminar ePTFE mesh. The focus of the study was on key events in adhesion formation such as mesothelialization of the mesh surface and collagen deposition triggered by implants. Collagen content evaluation, measured by immunohistochemical and gene expression analyses, served a dual purpose. On the one hand, collagen is indicative of the stabilization and maturation process undergone by the fibrin matrix, which constitutes the first step towards forming a permanent adhesion. On the other hand, it provides information regarding the degree of tissue incorporation of the mesh into the host tissue. Both of these are critical factors when evaluating the suitability and efficacy of a mesh for intraperitoneal use.

Sequential evaluation at 7 and 14 days permitted the adhesiogenic process to be tracked and correlationed with the subsequent studies performed in each animal.

## Materials and methods

### Meshes

**Fig 1:**
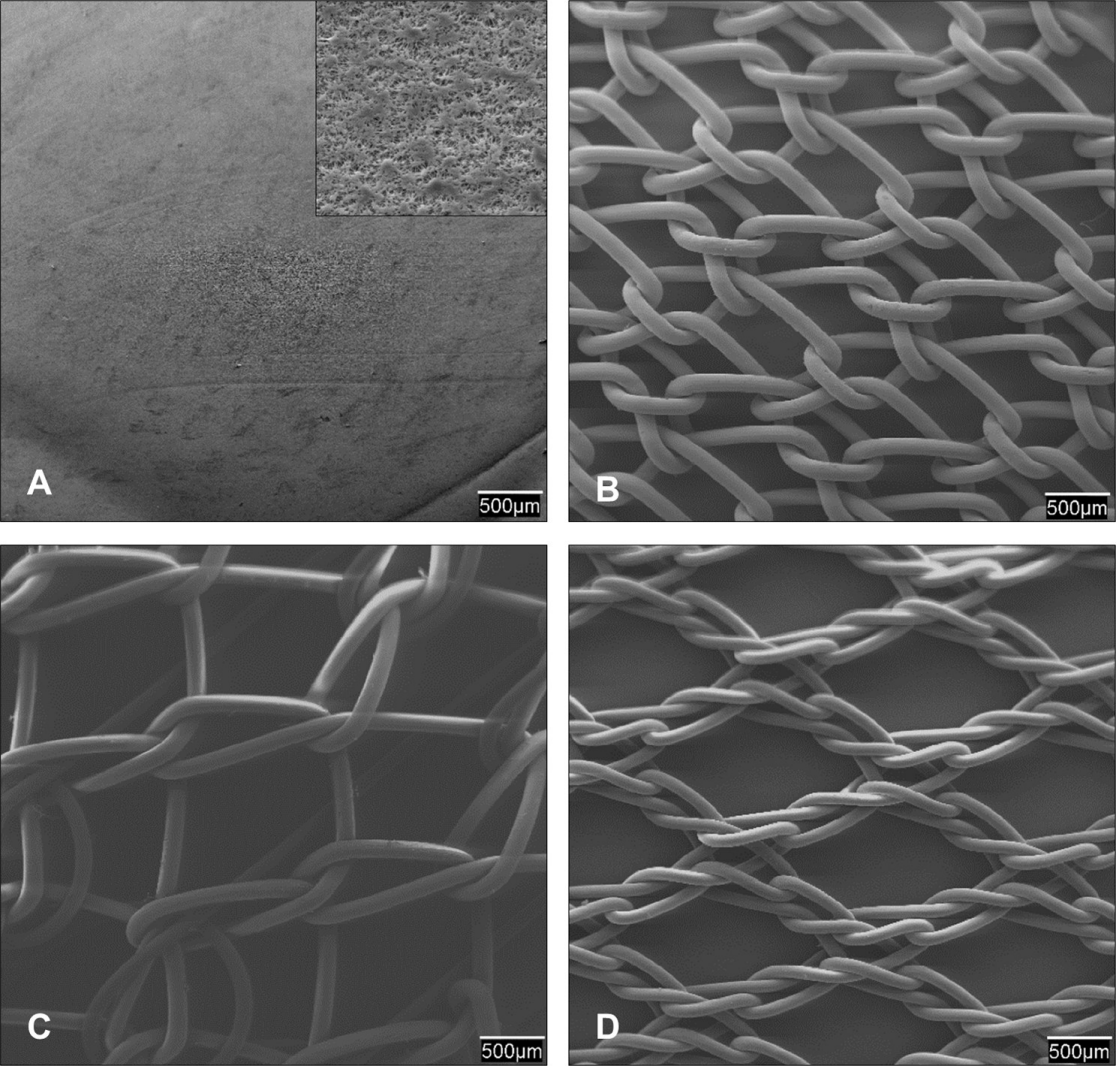
Characterization of the prosthetic materials. Scanning electron microscopy (SEM) images (20x magnification) of: (A) the laminar expanded polytetrafluoroethylene (ePTFE). A further magnified view (2000x) is shown in the box; (B) The bare reticular polypropylene alone (PP, Surgipro); (C) DynaMesh (PP interwoven with polyvinylidene fluoride, PVDF) and (D) TiMESH (PP coated with titanium). Note that for DynaMesh (C) the PVDF side (designed to be viscerally deployed) is shown.

The following prosthetic materials (Fig 1) were used for *in vitro* and *in vivo* experiments:

- Surgipro (Covidien, Mansfield, MA, USA): Polypropylene (PP) reticular mesh (84 g/m^2^). Tensile strength: 58.81 ± 7.21 N/cm (S1 Fig).
- DynaMesh IPOM (FEG Textiltechnik, Aachen, Germany): Monofilament 2-component structure of polyvinylidene fluoride (PVDF) fibers (88%, visceral side) interlinked with PP filaments (12%, parietal side). Effectively 108 g/m^2^ but corresponding to a conventional PP-mesh of approximately 60 g/m^2^, according to the manufacturer. Tensile strength: 56.29 ± 2.43 N/cm (S1 Fig).
- TiMESH (pfm medical, Köln, Germany): PP filaments coated with titanium (65 g/m^2^). Tensile strength: 20.14 ± 1.98 N/cm (S1 Fig).
- Preclude (Gore, W. L. Gore & Associates, Inc. Flagstaff, AZ, USA): Laminar expanded polytetrafluoroethylene (ePTFE) mesh (74 g/m^2^). Tensile strength: 26.69 ± 1.26 N/cm (S1 Fig.)

### *In vitro* study

#### Preparation of the prosthetic materials

**Fig 2:**
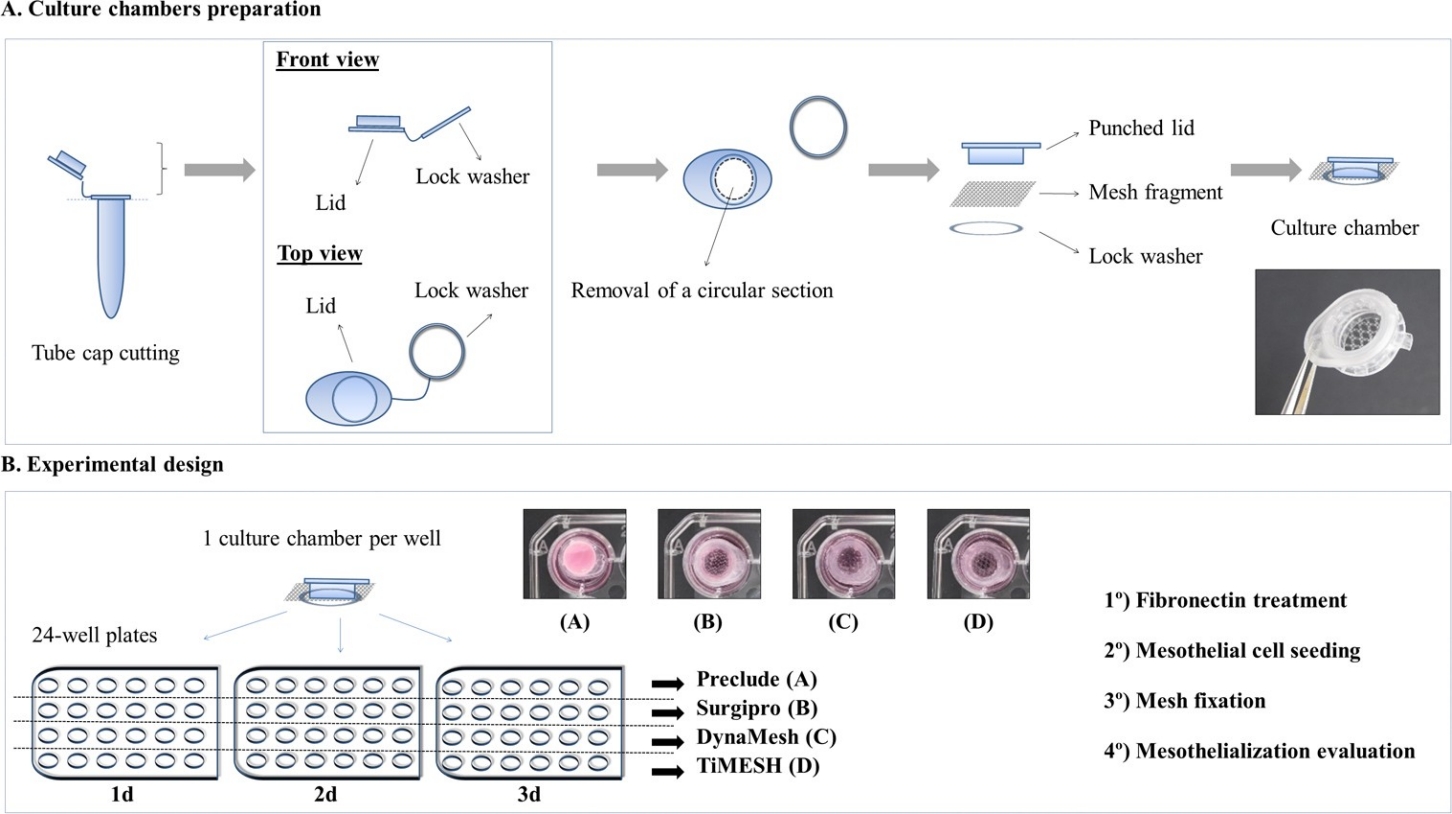
*In vitro* study. Illustration depicting the design of the culture chambers (A) and the experimental setup (B).

Under sterile conditions, the meshes were cut into 1 cm^2^ square-shaped fragments. Next, culture chambers were designed for each fragment using both the lid and the lock washer of sterile 1.5 mL Eppendorf tubes (Fig 2A). After cutting the tube caps, the lids were punched through to create a circular hole. Then, the mesh fragment was placed in direct contact with the inner surface of the punched lid, and the lock washer was utilized to secure the fragment to the lid margins. These devices were created to avoid mesh flotation during the cell seeding and culturing processes. Immediately prior to cell seeding, culture chambers (6 per study group) were placed into 24-well plates and treated with 20 μg/mL fibronectin (Sigma-Aldrich, St. Louis, MO, USA) for 1 h at 37°C (Fig 2B).

#### Mesothelial cell harvesting and culture

Mesothelial cells (MC) were isolated from mesenteric omental biopsies (20–25 g) collected from New Zealand white rabbits at the Animal Research Center of the University of Alcalá. To accomplish this in accordance with the animal welfare 3R´s criteria, the cells were obtained from biopsies from 3 euthanized rabbits that had previously been utilized in another study whose protocols were approved by the Committee on the Ethics of Animal Experiments of the University of Alcalá (registered code: OH-UAH2016/012). The fragments of omentum were processed under sterile conditions following a collagenase incubation standard protocol (S1 Protocol) performed by our group [20]. The cell precipitate was resuspended in 4 mL of low-glucose Dulbecco´s Modified Eagle´s Medium (DMEM) (Life Technologies Corporation) containing 10% fetal bovine serum (FBS) (Sigma-Aldrich) and 1% penicillin-streptomycin solution (Life Technologies Corporation). The MC were transferred to 25 cm^2^ culture flasks and incubated under controlled conditions (37°C, 5% CO_2_, humid atmosphere). Medium was changed every 3 days, and semiconfluent cultures were passaged at a 1:4 ratio. Cultures were visualized with a Zeiss Axiovert 40C phase-contrast microscope (Carl Zeiss, Oberkochen, Germany).

#### Cell seeding

Third-passage MC were transferred to fibronectin-treated culture chambers (Fig 2B) at a density of 2–2.5 x 10^5^ cells/well (S1 Protocol). Complete DMEM was added to each chamber to reach a final volume of 1 mL per well. The seeded meshes were incubated under controlled conditions and the MC adhesion was monitored using a phase-contrast microscope. After 24 h, 48 h and 72 hours, 3 fragments per study group were randomly selected, carefully washed in phosphate-buffered saline (PBS; pH 7.4) and fixed in 3% glutaraldehyde for further scanning electron microscopy (SEM) analysis.

#### Scanning electron microscopy

Samples were washed in Millonig buffer (pH 7.3) and dehydrated by incubation in an increasing graded ethanol series (30, 50, 70, 90 and 100%, 15 min each). Meshes were brought to the critical point in a Polaron CPD7501 desiccator (Fisons Instruments, Ipswich, UK), gold-palladium coated and visualized with a Zeiss DSM-950 scanning electron microscope (Carl Zeiss).

Non-seeded samples from the different study groups were also observed under SEM to assess the morphology of the biomaterials (Fig 1).

### *In vivo* preclinical study

The study protocol adhered to the ARRIVE (Animal Research: Reporting of *In Vivo* Experiments) guidelines for the publication of animal studies [21].

The study was carried out in strict accordance with the Guide for the Care and Use of Laboratory Animals of the National and European Institutes of Health (Spanish law 6/2013, Spanish Royal Decree 53/2013, European Directive 2010/63/UE and European Convention of the Council of Europe ETS123). All procedures were performed at the Animal Research Center of the University of Alcalá, which is registered with the Directorate General for Agriculture of the Ministry of Economy and Technology Innovation of the Community of Madrid (ES280050001165), indicating that all facilities legally cover the needs and requirements of the research. The study protocol (registered code: 2013/010/20130312) was approved by the Committee on the Ethics of Animal Experiments of the University of Alcalá.

#### Experimental design

Twenty-four male New Zealand white rabbits, weighing 3,000–3,400 g, were obtained from Granja Cunícola San Bernardo (Navarra, Spain) and quarantined for one week before the start of the study. Each animal was housed in a separate cage that consisted of two different compartments connected by a tunnel. They were kept under constant conditions of light and temperature, with *ad libitum* access to food and water allowed, and wood sticks as environmental enrichment devices. Animals were randomized to receive one of the following prosthetic materials: DynaMesh-IPOM (n = 6), TiMESH (n = 6), Surgipro (n = 6) or Preclude (n = 6). The adhesion formation process was tracked by laparoscopy at 7 days and animals were sacrificed at 14 days post-implantation for further analyses.

#### Surgical procedure and laparoscopy

All animals received 0.05 mg/kg buprenorphine (Buprecare, Divasa Farmavic, Barcelona, Spain) in their drinking water one hour before surgery as preemptive analgesia to guarantee its effect during the procedure and at the beginning of postoperative recovery. The animals were anesthetized by intramuscular injection with a mixture of ketamine (20 mg/kg, Imalgene 1000; Merial Laboratories, Barcelona, Spain) and xylazine (3 mg/kg, Xilagesic 2%; Calier Laboratories, Barcelona, Spain).

Using a sterile surgical technique, a longitudinal laparotomy (8 cm long incision) was performed along the midline. To the right of this incision, a prosthetic material fragment (5 x 3.5 cm) was placed on the intact parietal peritoneum and fixed at the corners and middle of both long sides by the placement of six transmural 4/0 polypropylene stitches (Tyco, Madrid, Spain). The abdominal wall was closed using a running 3/0 polypropylene suture, while the skin was closed by simple interrupted stitches with a 3/0 silk suture. Administration of 0.05 mg/kg buprenorphine in the drinking water was continued during the 3 following days to minimize pain.

Laparoscopy at 7 days post-implantation was performed under general anesthesia by introducing a 3 mm, 0° laparoscope (Karl Storz, Tuttlingen, Germany) into the peritoneal cavity through a 3.5 mm metal trocar (Karl Storz). An open technique was used to gain entry, making a 0.5–0.6 cm incision on the right side of the abdomen. To aid observations, the abdominal cavity was filled with CO_2_ at a maximum pressure of 8 mm of Hg. Observations were video recorded for subsequent review.

After 14 post-operative days, the animals were sedated with up to 20 mg/kg of xylazine (Rompun; Bayer, Leverkusen, Germany) and then euthanized with increasing concentrations of CO_2_ in a CO_2_ chamber. After CO_2_ inhalation, animals were monitored for the following signs: cessation of respiration and heartbeat, and pupillary dilation and unresponsiveness to light. Then, macroscopic images were taken and adhesion evaluation was performed for each animal. Implants, adhesions and omental tissue were collected for subsequent analysis at this time point after euthanasia in every case.

#### Adhesion evaluation

Areas of prosthetic materials covered by adhesions were determined at 7 days post-implantation using images captured by video recording during laparoscopy. Frontal images of the mesh were captured to have a view of the total area. Videos at a closer distance to the mesh were also recorded from different angles to confirm the extension of the tissue adhered, as well as to allow a clear visualization of the microvasculature in the adhesions. Adhesion outlines were transferred to a digital template of the mesh surface, producing an image that was analyzed using Image J (NIH, USA; http://imagej.nih.gov/ij). The results were expressed as the percentage area of the mesh surface occupied by adhesions scored as 0% (no adhesions) to 100% (mesh completely covered with adhesions). Adhesion classification was done according to laparoscopic observations as loose (transparent filmy adhesions with low or inexistent degree of vascularization), firm (dense and vascularized adhesions) or integrated (high degree of vascularization and penetration of the adhesion tissue through the mesh).

At the time of animal sacrifice 14 days post-implantation, adhesion percentage areas were quantified by tracing outlines of the adhesions on a transparent template of the same size as the mesh. These templates were subsequently digitalized. All the images were analyzed and results were expressed as previously described for 7-days laparoscopic observations.

Adhesions were evaluated and classified according to their macroscopic characteristics as: loose (transparent, poorly vascularized filmy adhesions that were easily dissected); firm, (denser adhesions, whitish in color and difficult to dissect); or integrated (requiring sharp dissection to pull away from the mesh, occasionally producing serosal damage of the organ involved).

#### Morphological analysis

After animal euthanasia at 14 days postsurgery, specimens were collected from the implants and omental adhesions to the mesh. Non-injured and adhesion-free omental tissue was also collected from each animal. For light microscopy, the specimens were fixed in F13 solution (60% ethanol, 20% methanol, 7% polyethylene glycol, and 13% distilled water), embedded in paraffin, cut into 5-μm-thick sections and subjected to hematoxylin-eosin, Masson’s trichrome (Goldner-Gabe) and Picrosirius red staining. These sections were then examined with a Zeiss Axiophot light microscope (Carl Zeiss, Oberkochen, Germany). Picrosirius red staining was also observed under polarized light, which allowed observation of collagen levels and different collagen fibers orientations.

Specimens (approximately 1 cm^2^) consisting of the mesh and the underlying abdominal wall were fixed in 3% glutaraldehyde and processed as previously described in the scanning electron microscopy section.

#### Immunohistochemistry

For immunohistochemical analysis (S2 Protocol), paraffin-embedded sections were equilibrated in phosphate-buffered saline (PBS; pH 7.4) and incubated with mouse monoclonal antibodies against collagen I COL-1 (ab6308; Abcam, Cambridge, UK) (1:100), collagen III hCL(III) clone III-53 (AF 5850; Medicorp, Montreal, Canada) (1:500) and the mouse monoclonal antibody against rabbit macrophages RAM-11 (M-633; Dako, Glostrup, Denmark) (1:50). The antigen-antibody reaction was detected by the alkaline phosphatase-labeled avidin-biotin procedure. Cell nuclei were counterstained for 5 minutes in Carazzi´s hematoxylin. Tissue sections were examined under a light microscope (Carl Zeiss, Oberkochen, Germany).

Quantification of RAM-11 positive cells was performed in 25 light microscopy fields (200x magnification) per sample in all groups by two independent observers in a blinded fashion.

#### Real-time reverse transcription-polymerase chain reaction (qRT-PCR)

Fragments of the implants (1 cm^2^ in size), omental adhesion tissue and non-injured omentum were obtained and stored at -80°C until further analysis (S3 Protocol).

RNA was extracted through guanidine-phenol-chloroform isothiocyanate extraction procedures with TRIzol (Invitrogen, Carlsbad, CA, USA). RNA amounts and purity were measured at an optical density of 260/280 nm and 260/230 nm in a NanoDrop ND-1000 spectrophotometer (Thermo Fisher Scientific Inc., DE, USA) while RNA integrity was checked using 1% (wt/vol) agarose gel electrophoresis.

Complementary DNA was synthesized from 200 ng of total RNA by reverse transcription (RT) using oligo dT primers (Amersham, Fairfield, USA) and the M-MLV reverse transcriptase enzyme (Invitrogen).

Complementary DNAs (cDNA) were amplified using the following primers: collagen 1A2 (col 1) (sense 5´-ATG GTG GCA CCC AGT TTG AA -3´ and antisense 5´-AGG TGA TGT TCT GAG AGG CG -3´), collagen 3A1 (col 3) (sense 5´-TGC TAA GGG TGA AGT TGG AC -3´ and antisense 5´-CCG CCA GGA CTA CCA TTG TT -3´) and GAPDH (sense 5´-TCA CCA TCT TCC AGG AGC GA-3´ and antisense 5´-CAC AAT GCC GAA GTG GTC GT-3´).

The RT-PCR mixture contained 5 μl of the inverse transcription product (cDNA) diluted 1:20, 10 μl of iQ SYBR Green Supermix (Bio-Rad Laboratories, Hercules, CA, USA), 1 μl (6 μM) of each primer (sense and antisense) and 3 μl of RNase-free water for a final reaction volume of 20 μl. RT-PCR was performed in a StepOnePlus Real-Time PCR System (Applied Biosystems, Foster City, California, USA). The samples were analyzed in triplicate and gene expression was normalized against the expression value recorded for the constitutive gene glyceraldehyde 3-phosphate dehydrogenase (GAPDH).

#### Statistical analysis

The data were expressed as the means ± standard error of the mean (SEM), and all the statistical tests were performed using the program GraphPad Prism 5 computer package (GraphPad Software, Inc., La Jolla, CA, USA) for Windows.

Adhesion percentages were subjected to one-way analysis of variance testing (ANOVA). When this test was positive, the Tukey´s test for pairwise comparison of subgroups was used. Macrophage counts and collagen mRNA expression levels were compared between pairs of groups using the Mann-Whitney U test.

The level of significance was set at p<0.05 in all cases.

## Results

### *In vitro* study

#### Mesothelial cell coverage

Harvested mesothelial cells from omental biopsies displayed polygonal shapes and growth in a cobblestone-like monolayer, typical characteristics of this cell lineage.

**Fig 3:**
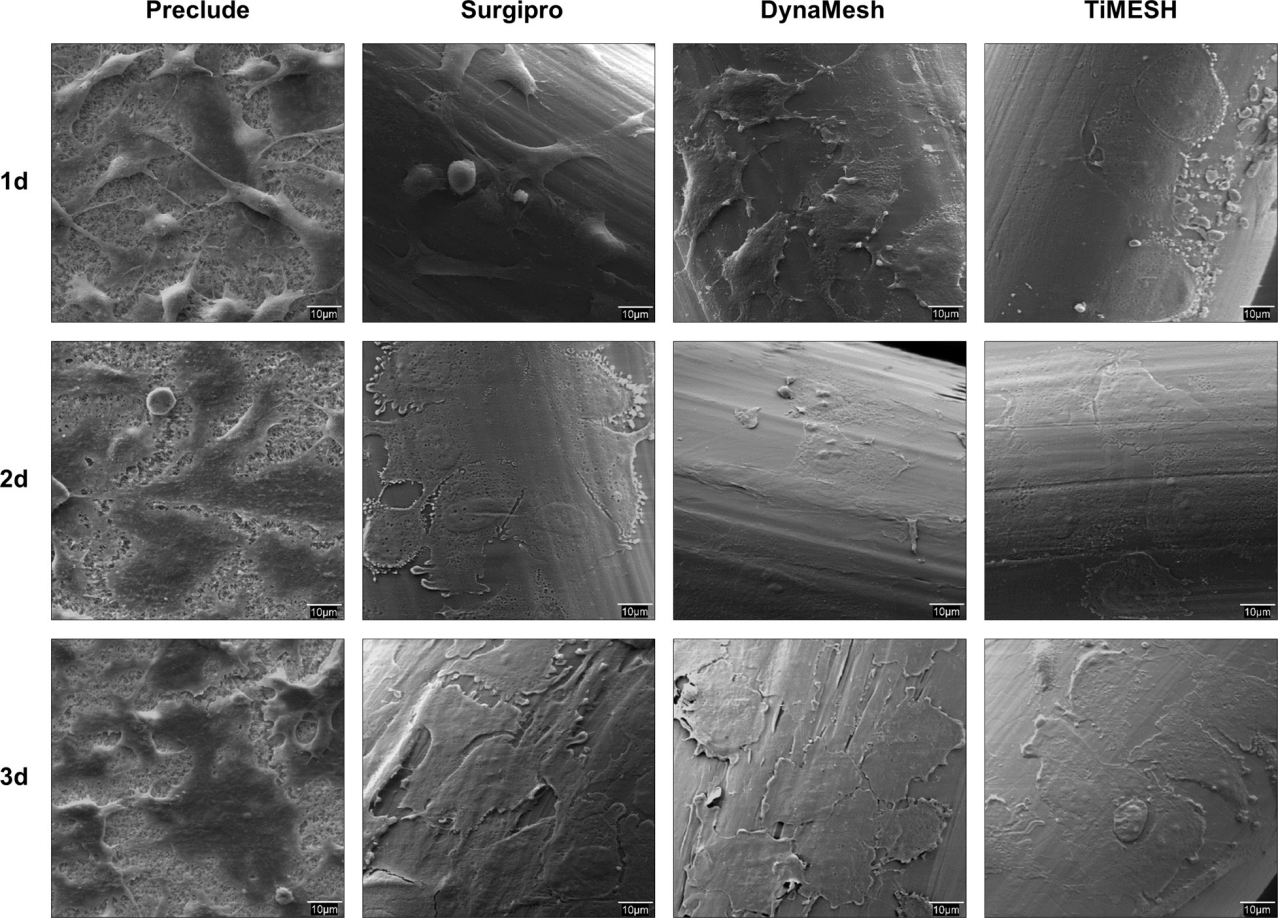
*In vitro* mesothelialization of meshes. Scanning electron microscopy (1000x magnification) of the mesh fragments seeded with omental mesothelial cells. The micrographs are representative of every study group: Preclude (laminar e-PTFE); Surgipro (bare reticular PP alone), DynaMesh (PP interwoven with PVDF) and TiMESH (PP coated with titanium) at 1, 2 and 3 days after seeding.

The behavior of the seeded reticular meshes (Surgipro, DynaMesh or TiMESH) both in culture and in SEM analysis (Fig 3) was consistently similar regardless of their composition (PP, PP interwoven with PVDF or PP coated with titanium, respectively). Following 1 day of seeding, the mesh filaments were partially covered with several and different-sized MC colonies. An increase in the cell coverage was observed over time, although a cell monolayer had not been fully developed at the final 3-day time point. Moreover, the mesh pores were devoid of cells, given the lack of solid substrates in those regions.

Compared to the reticular meshes, the laminar ePTFE material (Preclude) showed greater cell coverage over the mesh area. By day 1, most of the MC were spread over the mesh and displayed a markedly proliferative status, as evidenced by a high number of cells undergoing mitosis. By days 2 and 3, a cell monolayer was visible, showing mild denuded areas dispersed along the surface.

### *In vivo* preclinical study

There was no mortality in any of the study groups, with all the animals reaching the final study time point set at 14 days and ultimately being included in all of the analyses. None of the animals exhibited postoperative complications and no behavioral signs implying a decrease in animal welfare were observed.

#### Laparoscopy, macroscopic observations and adhesion coverage

**Fig 4:**
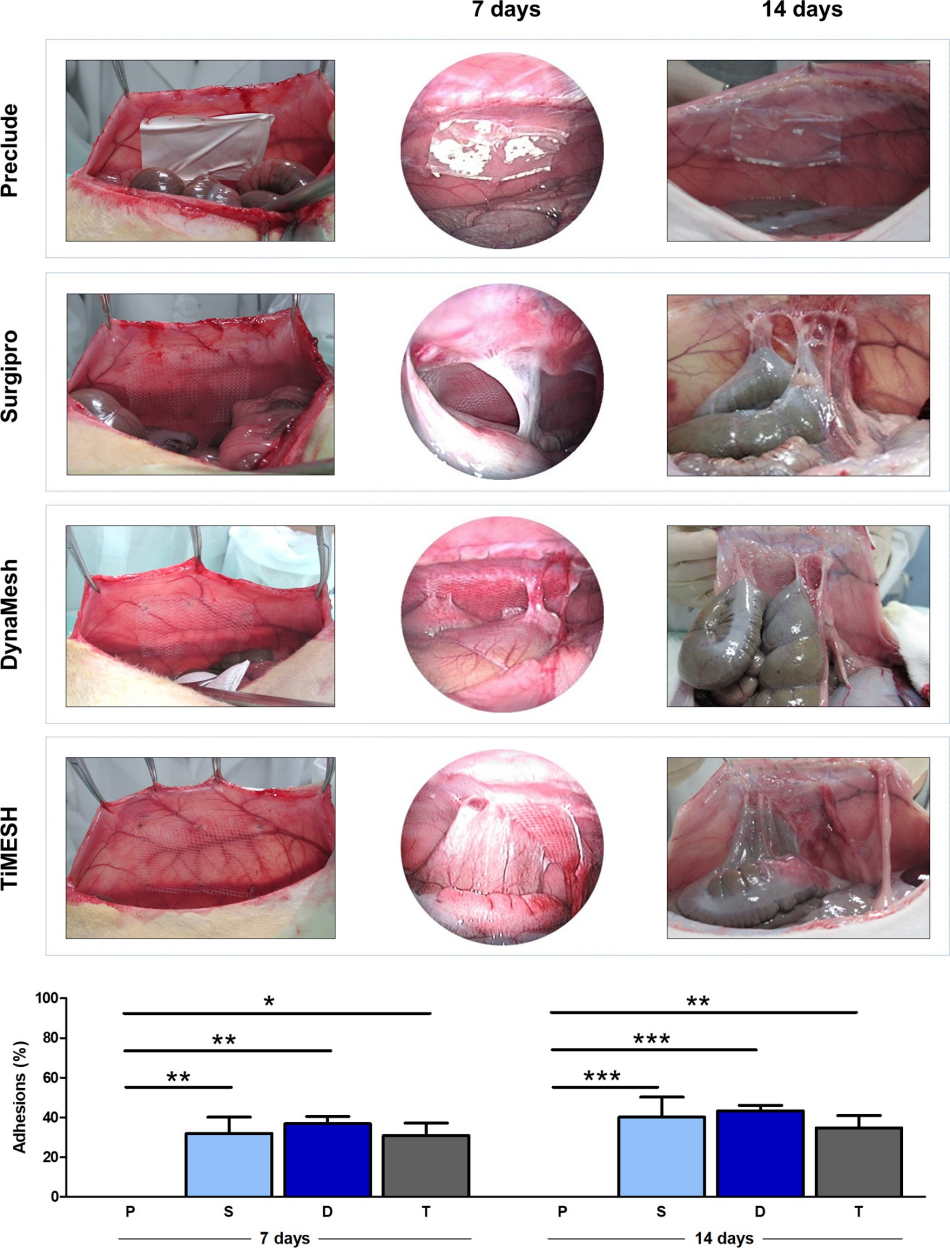
Surgical technique, laparoscopy and adhesion formation. Macroscopic appearance of the meshes after implantation and laparoscopic analysis in the different experimental groups: Preclude (P, laminar e-PTFE); Surgipro (S, bare reticular PP alone) and the two hybrid reticular meshes: DynaMesh (D, PP interwoven with PVDF) and TiMESH (T, PP coated with titanium). Percentage of the mesh areas covered by adhesions for each experimental group at 7 and 14 days post-implantation are shown. Reticular meshes (Surgipro, DynaMesh and TiMESH) showed no differences between each other at any study time point, but differences were significantly higher for all of them compared to the laminar Preclude (*p<0.05, **p<0.01, ***p<0.001).

Preclude (laminar ePTFE) implants showed no adhesions at any of the follow-up time points analyzed (Fig 4). This mesh showed deficient integration into the host tissue but a slight adhesion to the peritoneal surface, favouring that the material maintained its initial position and no displacement occurred.

All of the reticular meshes (Surgipro, DynaMesh and TiMESH) showed comparable behavior, with adhesions established 7 days post-implantation that remained at 14 days (Fig 4). Adhesions were of the firm or integrated type affecting the omentum and the intestinal loops, with no loose adhesions at the established follow-up times or noticeable differences among these groups in the severity of the adhesions formed. Omental adhesions were intensely vascularized, and no cases of bowel strangulation were reported.

Macroscopic findings showed excellent incorporation of the reticular meshes into the abdominal wall regardless of their composition. The quantitative analysis of the mesh surface areas covered by adhesions (Fig 4) revealed similar adhesion percentages for Surgipro (S), DynaMesh (D) and TiMESH (T), with significant differences found only when reticular meshes were compared to the laminar material Preclude (P), at both 7 (p<0.05, Preclude vs. TiMESH; p<0.01, Preclude vs. Surgipro, DynaMesh) and 14 days (p<0.01, Preclude vs. TiMESH; p<0.001, Preclude vs. Surgipro, DynaMesh). An increase in the percentage adhesion score over time was identified for all reticular meshes, although the difference was not statistically significant.

#### Morphological assessment of implants

**Fig 5:**
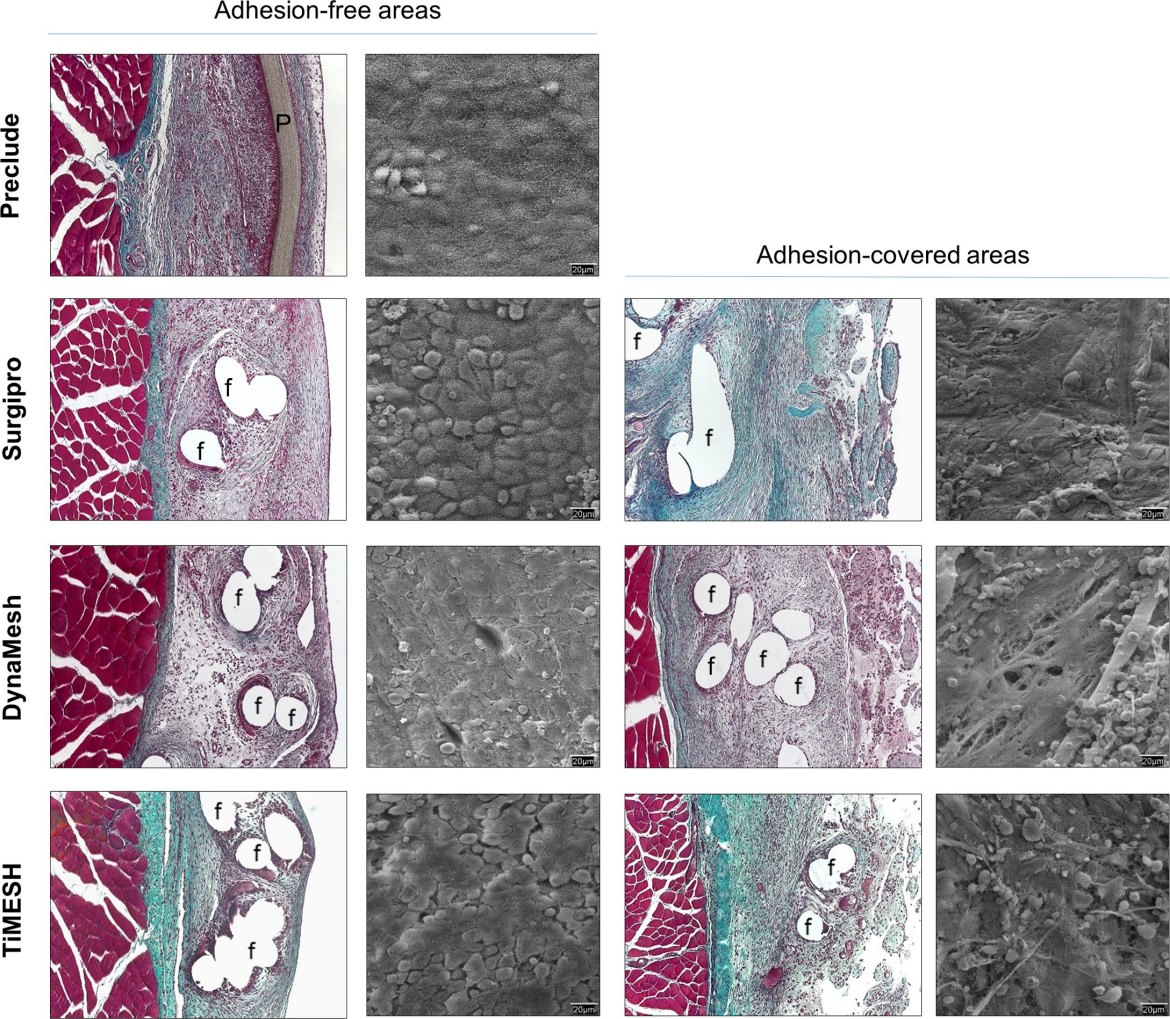
Morphological studies of the implanted meshes. Light microscopy (Masson´s trichrome staining, 100x magnification) and scanning electron microscopy micrographs of the surface of the materials (500x magnification) in adhesion-free and adhesion-covered areas for each group at 14 days post-implantation. The Preclude group showed no adhesion-covered areas in any animal. f: mesh filaments, P: Preclude.

The lack of pores in the Preclude limited biomaterial incorporation into the host tissue, showing partial encapsulation. The transition zone between this capsule and the recipient abdominal wall was highly cellular and showed a compact collagen matrix formed by collagen bundles running parallel to the longitudinal axis of the mesh in only the inner third of the parietal peritoneum. The neoperitoneum exhibited numerous small- and medium-sized blood vessels as well as a continuous flat, smooth-surfaced typical mesothelial monolayer on the visceral side towards the abdominal cavity, as demonstrated by SEM (Fig 5).

The excellent integration of PP-based reticular meshes (Surgipro, DynaMesh and TiMESH) in the host tissue was confirmed by histological analysis (Fig 5) and was similar among each of them. Newly formed connective tissue appeared to completely fill interfilament spaces, giving rise to optimal incorporation of the mesh into the abdominal wall. All of these meshes showed a substantial presence of white blood cells, especially at the neoformed tissue in the subcutaneous side of the parietal peritoneum, together with fibroblastic cells in the proximity of the prosthetic filaments. Loose collagen fibers were observed in granulation tissue areas. Adhesion-free sectors showed the presence of a monolayer composed of rounded mesothelial cells with secretory features on the SEM analysis (Fig 5), with few areas of stable mesothelium. Those areas covered with adhesions showed scarce patches of disrupted mesothelium and exposure of the extracellular matrix of the submesothelium, where a large number of white and red blood cells had been attached.

#### Collagen expression in implants

**Fig 6:**
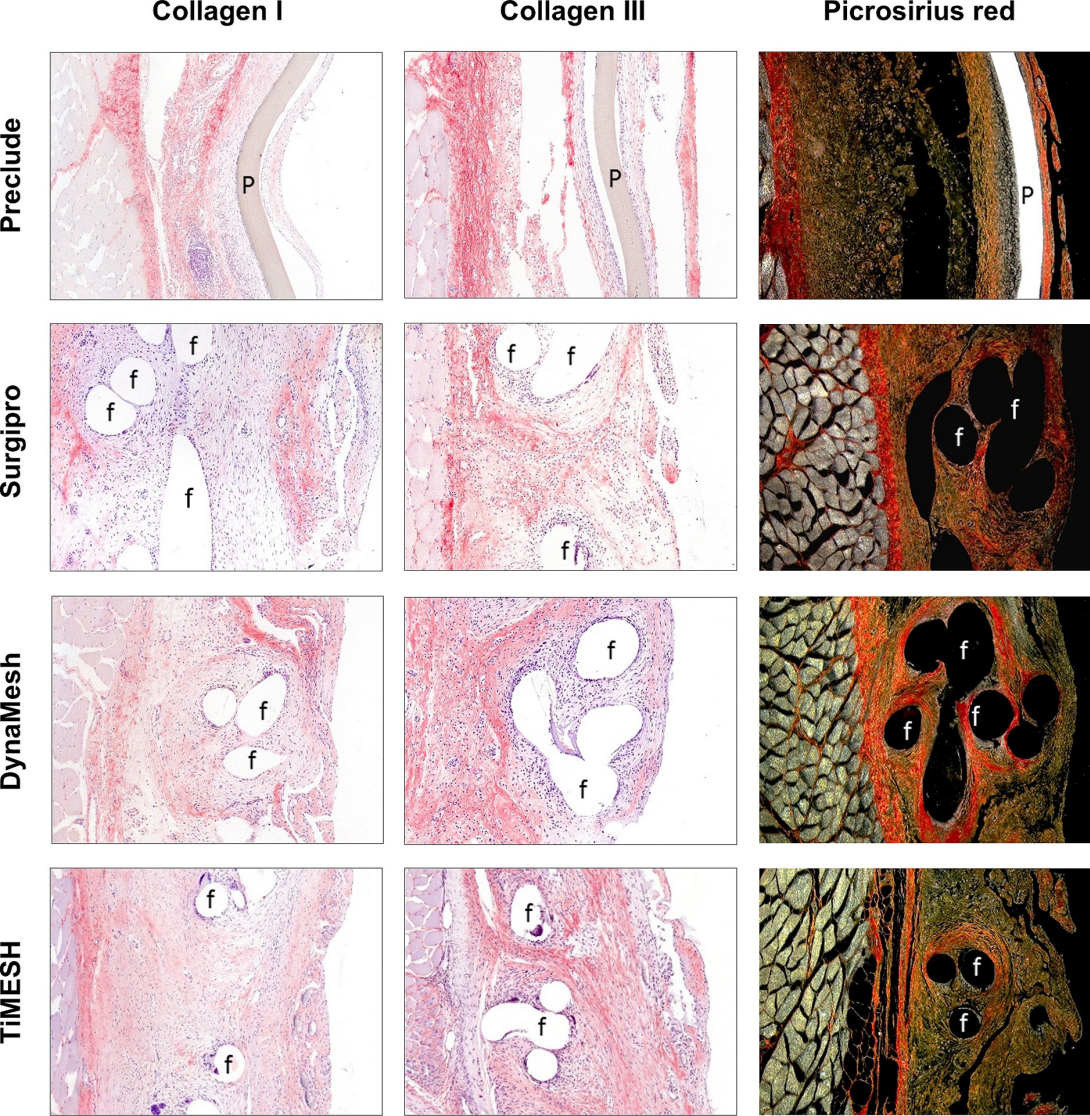
Collagen expression. Immunolabeling for collagens type I and III and Picrosirius red staining in implants from the different study groups (100x magnification). f: mesh filaments, P: Preclude.

In the laminar mesh Preclude, collagen type III, whose expression in the neoperitoneum was more extensive than that of collagen type I, appeared as organized collagen fibers laying parallel to the abdominal wall in the inner third while was distributed in a looser disposition in closer proximity to the mesh. Vast areas covered by an inflammatory cell infiltrate were observed in the mesh/subcutaneous tissue interface, where collagen content was practically null. Collagen type I appeared in the inner zone of the parietal peritoneum and particularly in extensive microvascular areas as a loose network. Picrosirius red staining permitted demonstration of the limited collagen presence at the mesh-subcutaneous tissue interface, which accounts for the lack of integration of this mesh (Fig 6).

Collagen type III fibers were observed concentrically surrounding the polypropylene filaments in Surgipro and in areas rich in inflammatory cells, mainly in a loose reticular organization rather than in dense fibers. By contrast, collagen type I was not associated with filaments of PP but with the interfilament spaces. Its presence was mainly found close to the subcutaneous tissue and on the visceral side of the neoperitoneum (Fig 6).

For TiMESH and DynaMesh, a similar deposition and distribution pattern for both types of collagen was identified, with the predominance of collagen type III over collagen type I (Fig 6). Collagen type III was deposited around the mesh filaments and in the neoperitoneum mainly as a loose fibrillar network. Significant expression was also manifested in the frequent areas of granulation tissue located in the mesh-host subcutaneous tissue interface. Collagen type I was present in microvascular zones of the parietal neoperitoneum and, as in Surgipro, in the spaces between filaments instead of in close contact with them.

Picrosirius red staining (Fig 6) revealed high collagen levels in the neoformed tissue and heterogeneity in the orientation of collagen fibers for all reticular meshes studied. In general, collagen fibers comprising the connective tissue capsule that enveloped groups of filaments displayed a staining pattern similar to fibers in the native fascia. By contrast, the matrix in the neoperitoneum in those areas away from filaments showed poorer organization and different orientation.

#### Macrophage presence in implants

**Fig 7:**
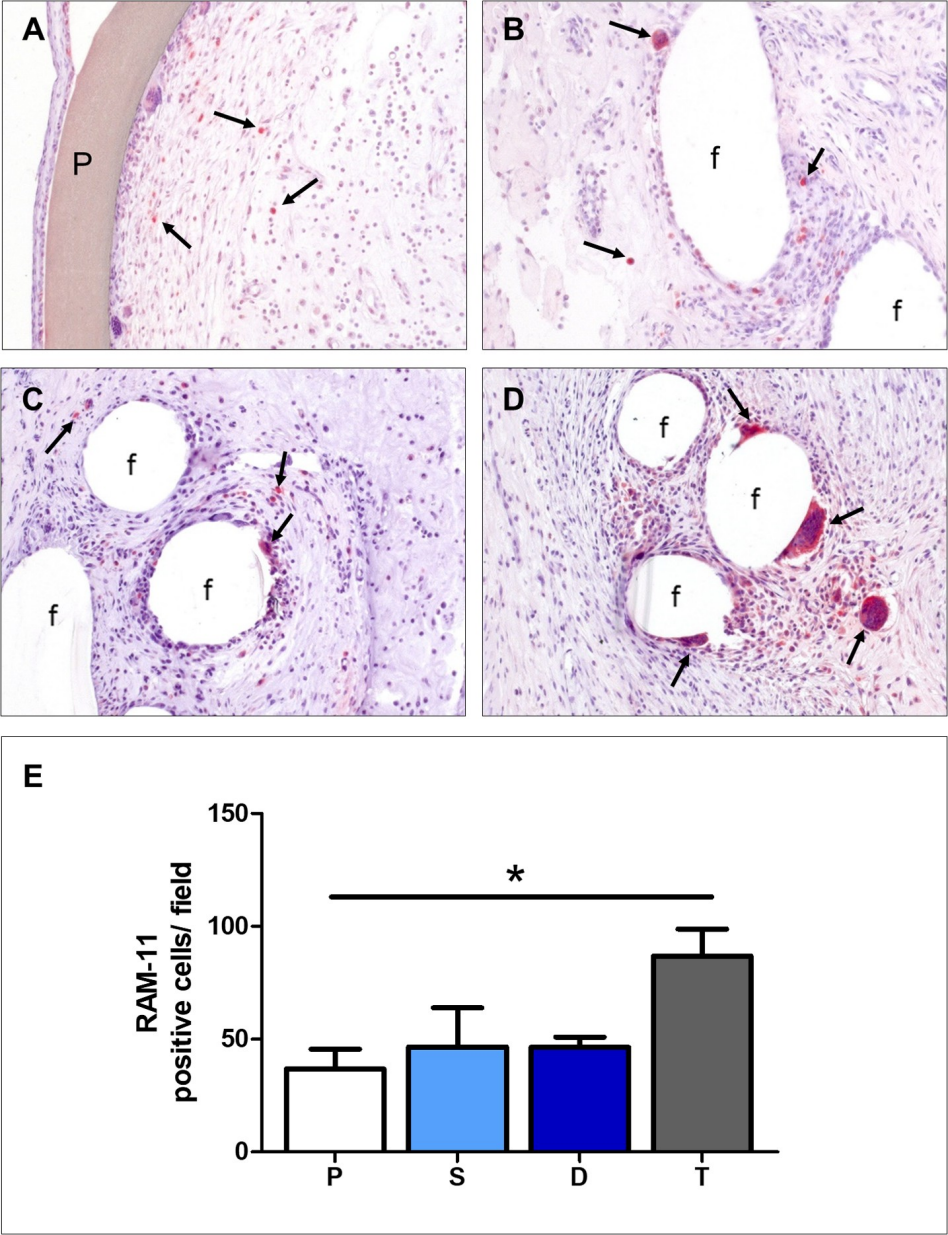
Foreign-body reaction of the different meshes. RAM-11 monoclonal antibody immunohistochemical staining showing the presence of labeled macrophages (→) in proximity to the different mesh materials (200x magnification): (A) Preclude, (B) Surgipro, (C) DynaMesh and (D) TiMESH. f: mesh filaments, P: Preclude. (E) Positive cell counts per field recorded after 14 days of implantation. The results are shown as the means ± standard error of the mean for the different study groups: P (Preclude), S (Surgipro), D (DynaMesh) and T (TiMESH). *p<0.05.

The maximal presence of macrophages was noted in TiMESH implants (Fig 7D), although the difference with the two other PP-based reticular meshes (Fig 7B and 7C) was statistically non-significant (Fig 7E). Significant differences emerged when TiMESH was compared to the Preclude implant (p<0.05) (Fig 7A and 7E).

Inflammatory reactions were limited to the perifilamentary region with reticular meshes and lined the biomaterial in the laminar mesh Preclude. Multinucleated foreign-body giant cells were mainly observed in the TiMESH group, in close proximity to the material filaments.

#### qRT-PCR in implants

**Fig 8:**
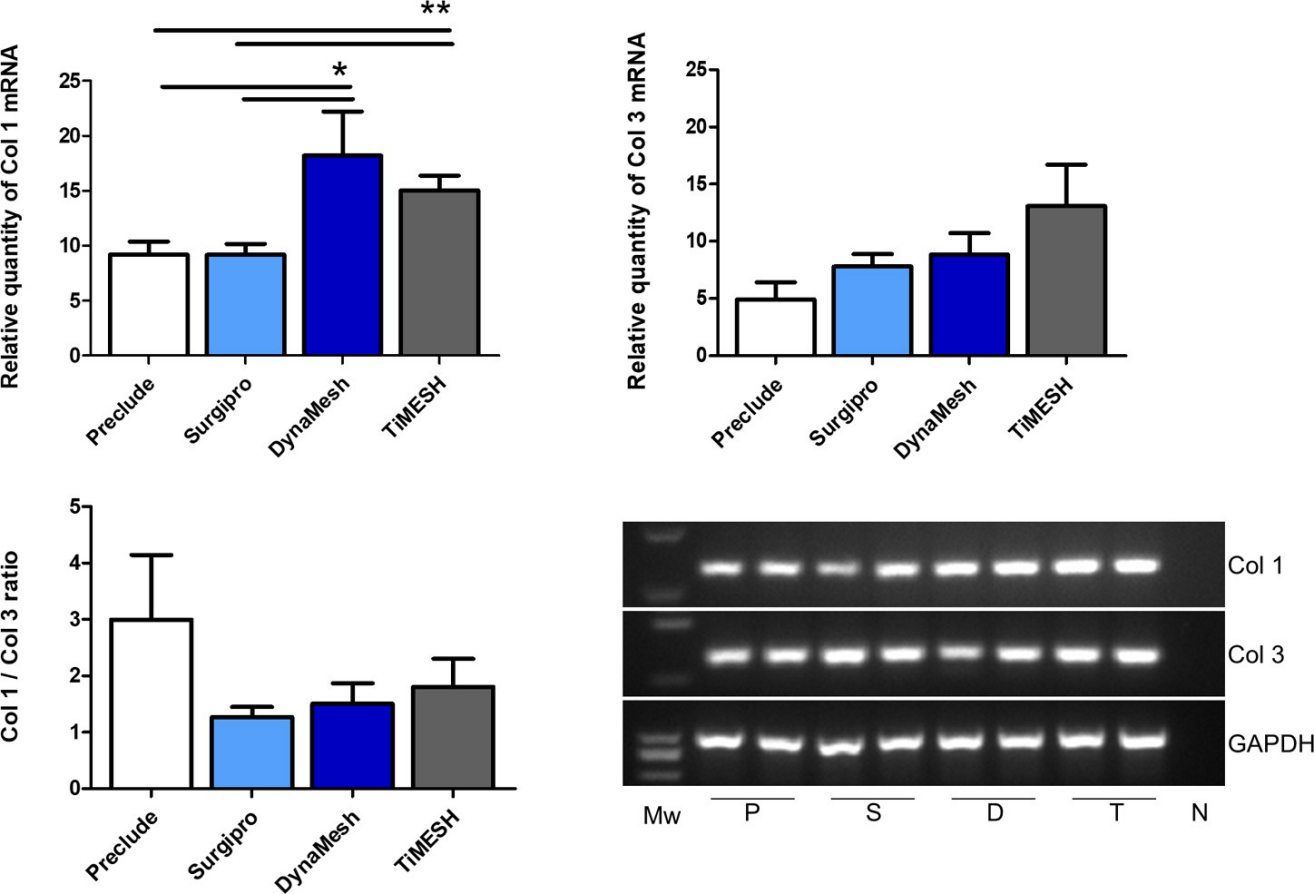
Collagen 1 and 3 mRNA expression levels determined by RT-PCR in the implant areas. Relative mRNA levels of collagen 1, collagen 3 and the Col1/Col 3 ratio in implant samples from the different experimental groups. Gene expression was normalized to the expression recorded for the reference gene GAPDH. Agarose gel electrophoresis of RT-PCR products is shown. Each pair of lanes is representative of each experimental group: P, Preclude; S, Surgipro; D, DynaMesh; T, TiMESH. Mw: Molecular weight markers. N: Negative. Collagen 1 mRNA expression was statistically greater in the hybrid meshes DynaMesh (*, p<0.05) and TiMESH (**, p<0.01) compared to Surgipro and Preclude. No differences were found in collagen 3 mRNA expression levels or the Col1/Col3 ratio.

Collagen 1 mRNA showed a higher expression level for both hybrid meshes DynaMesh (p<0.05) and TiMESH (p<0.01) compared to Preclude and Surgipro (Fig 8).

Collagen 3 mRNA expression showed the lowest levels with Preclude and the highest with TiMESH, although the differences did not reach statistical significance. When the Col1/Col 3 ratio was calculated, the highest value was found for Preclude. Differences, however, were not statistically significant between any of the groups for this ratio (Fig 8).

#### Morphology and collagen levels in adhesion tissues

**Fig 9:**
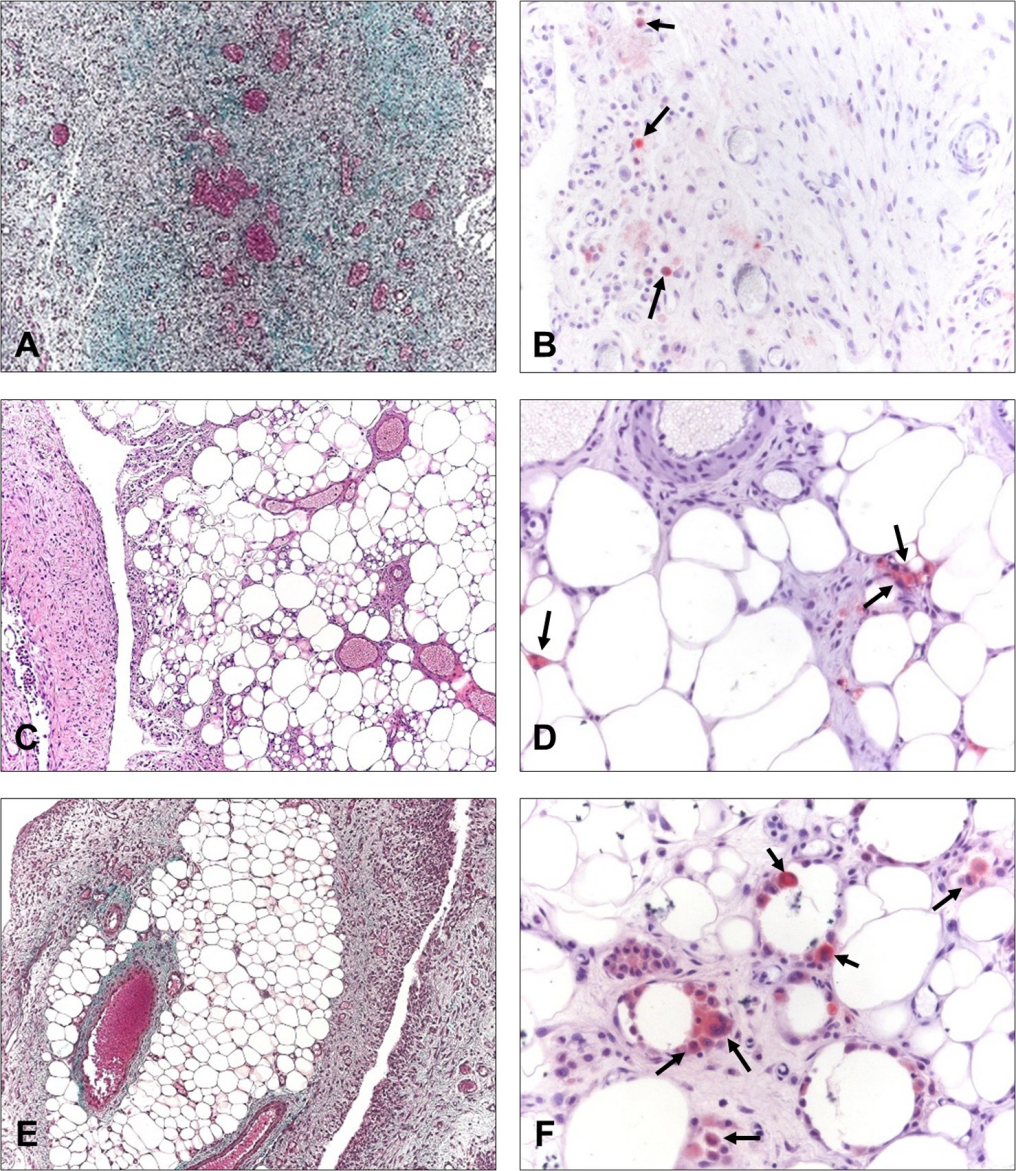
Morphological studies and foreign-body reaction in adhesion tissue. Representative light microscopy micrographs (A and E, Masson´s trichrome staining, 100x magnification; C, hematoxylin-eosin staining, 100x magnification) and immunohistochemical labeling of rabbit macrophages (→) using the RAM-11 monoclonal antibody (B, D and F, 320x magnification) in adhesion tissue formed to the different meshes (A and B, Surgipro; C and D, DynaMesh; E and F, TiMESH).

**Fig 10:**
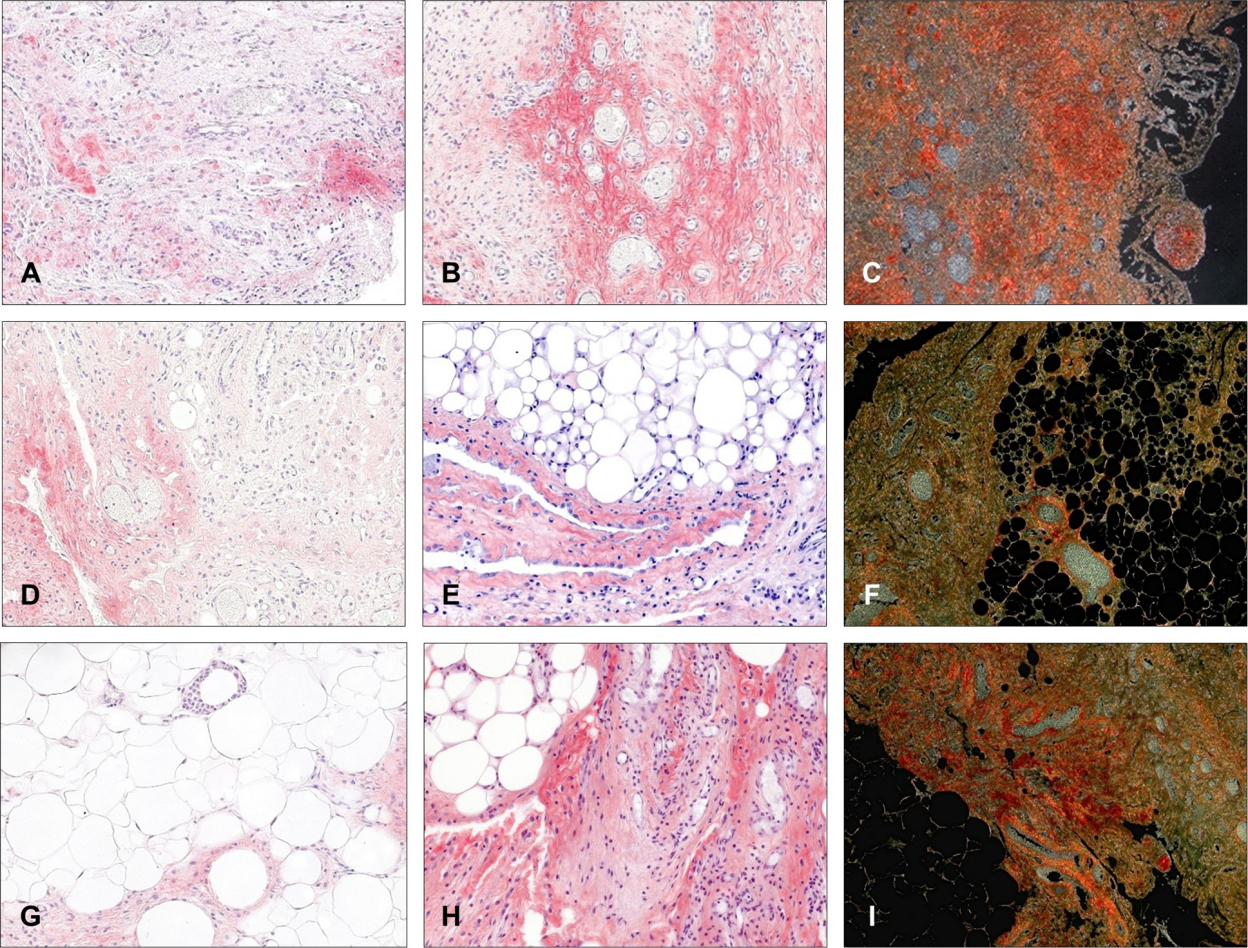
Collagen expression in adhesion tissue. Immunohistochemical labeling for collagen type I (A, D and G, 200x magnification), collagen type III (B, E and H, 200x magnification), and Picrosirius red staining (C, F and I, 100x magnification) in adhesion tissues from animals implanted with different meshes (A, B and C, Surgipro; D, E and F, DynaMesh; G, H and I, TiMESH).

In the Surgipro group, adhesions appeared to be mostly comprised of a highly cellular compact fibrous tissue (Fig 9A) with a predominance of collagen type III (Fig 10B), especially in the submesothelial layer and in microvascular areas. In general, collagen type I staining (Fig 10A) was weak and diffuse, exhibiting its greatest intensity in zones with the presence of small neovessels. Those areas rich in cells with myofibroblastic appearances were negative for both types of collagen.

Adhesion tissue in the DynaMesh group showed the highest preponderance of adipose composition among all the meshes studied (Fig 9C). Zones of adipose regeneration could be seen, with smaller adipocytes and a considerable presence of inflammatory cells. Collagen type I staining (Fig 10D) was scarce and heterogeneously distributed, generally restricted to the extracellular matrix around vessels, deposits in areas of minimal cellularity and small patches in the submesothelial stroma. Similarly, collagen type III could also be found in the submesothelial stroma (Fig 10E) and occasionally in microvascular regions, showing its distribution in bundles of collagen fibers.

When adhesions from the TiMESH group were studied, morphological heterogeneity was evidenced (Fig 9E). Several of the adhesions were mainly comprised of adipose, while others showed a prevalence of a fibrotic phenotype. Independent of the primary composition type, both phenotypes showed collagen type III in the submesothelial layer and in the vicinity of milky spots. Milky spots in these samples were numerous and displayed substantial cellular proliferation. Collagen type I expression was identified in specific limited areas in those adhesions of fibrotic morphology, while it was practically negative in those of primarily adipose composition (Fig 10G).

Picrosirius red staining showed the colocalization of collagen fibers with different orientations in those fibrotic intensely vascularized and cellular areas in the Surgipro group (Fig 10C). By contrast, in the TiMESH and the DynaMesh groups, areas with different orientations of the collagen fibers usually appeared exclusively (Fig 9F and 9I).

#### Macrophage presence in adhesion tissue

The presence of macrophages in adhesion tissue in the Surgipro group was scarce, only showing RAM-11- positive cells dispersed in several areas rich in inflammatory cells (Fig 9B). Adhesions in the DynaMesh group showed a higher proportion of macrophages, not only as isolated positive cells in fibrotic areas but also immersed in the adipose tissue, mainly as foreign-body giant cells or as part of milky spots (Fig 9D). As in the TiMESH implants, adhesion tissue from the group implanted with this mesh manifested a more intense foreign body reaction. Frequent foreign-body giant cells were observed, mostly related to activated milky spots (Fig 9F). These structures appeared interspersed in the adipose tissue, showing evident signs of cellular proliferation and colonization of the surrounding tissue by the cells comprising them.

#### qRT-PCR in adhesion tissue

**Fig 11:**
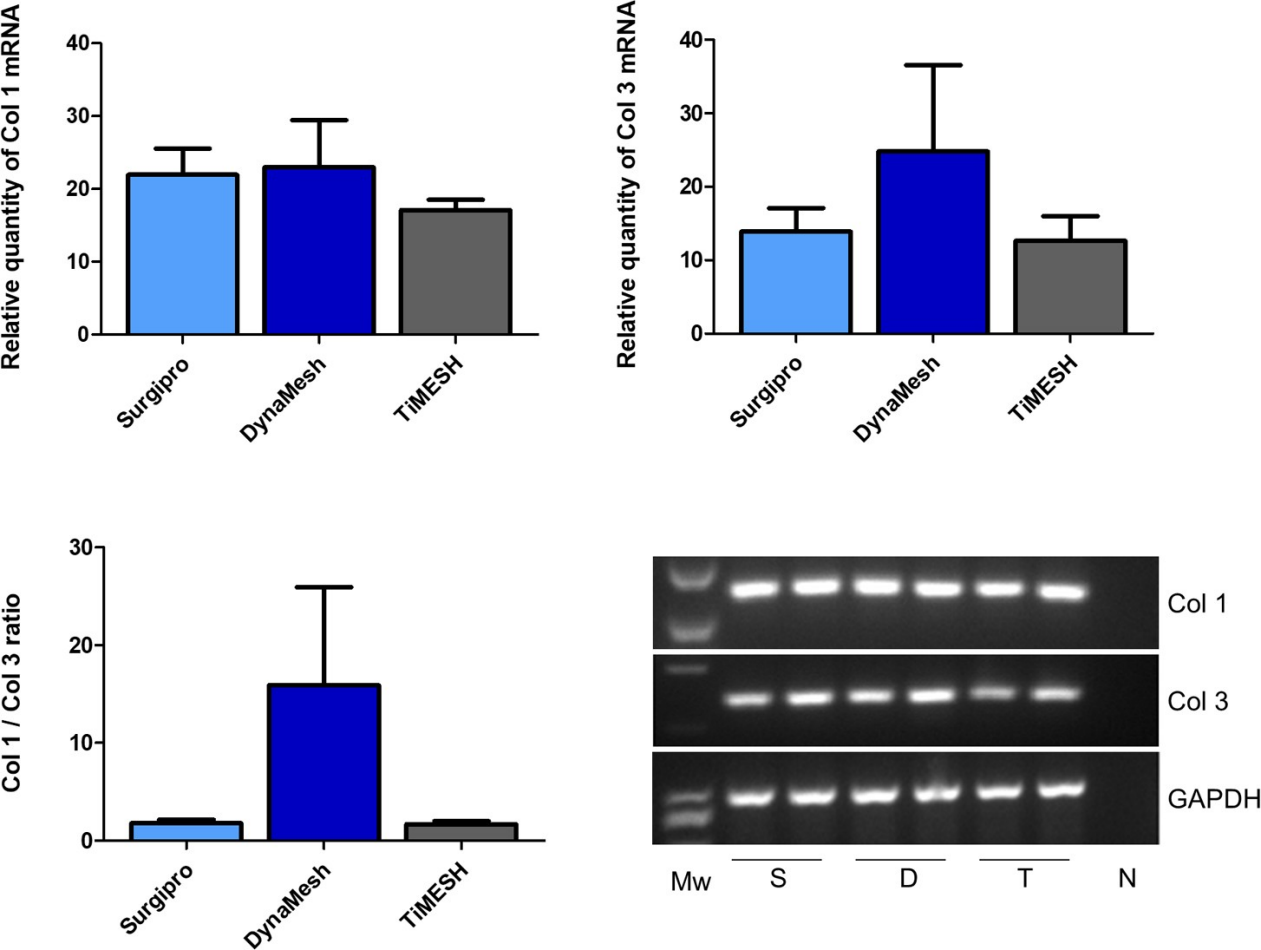
Collagen 1 and 3 mRNA expression levels determined by RT-PCR in adhesion tissue. Relative mRNA levels of collagen 1, collagen 3 and Col1/Col 3 ratios in adhesion tissues from the different experimental groups that developed adhesions (Surgipro, DynaMesh and TiMESH). Gene expression was normalized to the expression recorded for the reference gene GAPDH. Agarose gel electrophoresis of RT-PCR products is shown: S, Surgipro; D, DynaMesh; T, TiMESH. Mw: Molecular weight markers. N: Negative. No significant differences were found for collagen 1 or collagen 3 mRNA expression or for the Col 1/Col 3 ratios.

All the reticular meshes implanted induced similar levels of collagen 1 and collagen 3 mRNA in the adhesion tissue (Fig 11).

#### Omentum

**Fig 12:**
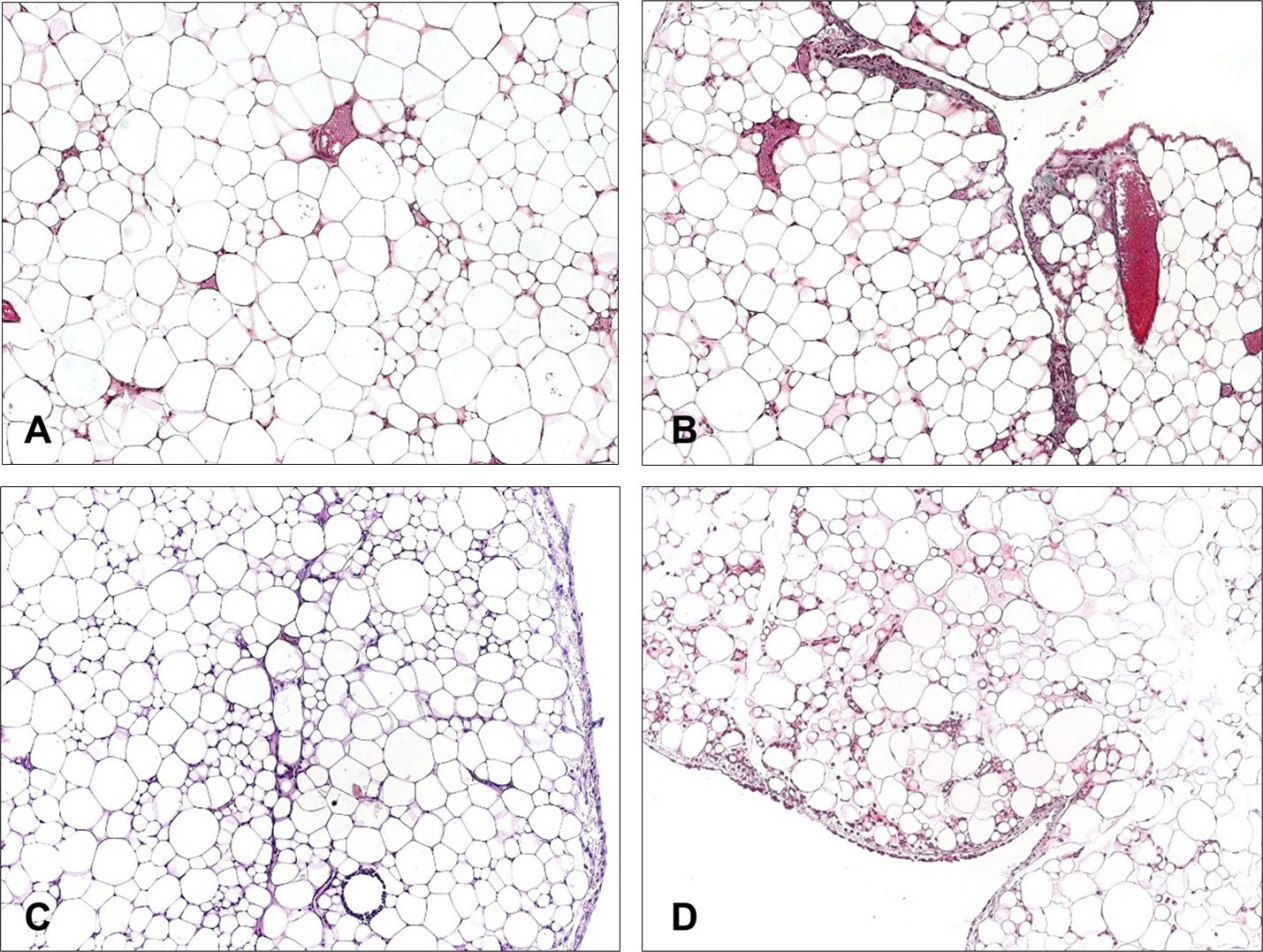
Morphological studies in non-injured omentum. Light microscopy micrographs (A, B and D, Masson´s trichrome staining, 100x magnification; C, hematoxylin-eosin, 100x magnification) of samples of omentum (non-injured and not involved in adhesions) from animals implanted with different meshes: (A) Preclude, (B) Surgipro, (C) DynaMesh and (D) TiMESH.

The omentum appeared as mature white adipose tissue divided by thin connective tissue septa, with the presence of vessels of different diameters and enclosed by a mesothelial monolayer (Fig 12).

In several areas, the presence of inflammatory cells together with preadipocytes distributed along the mature adipose tissue or associated with the submesothelial stroma was noted, which is compatible with zones of adipose tissue regeneration. Scarce RAM-11- positive cells were observed in the mesothelial-submesothelial layer of this tissue or in association with milky spots in all study groups.

#### qRT-PCR in omentum

**Fig 13:**
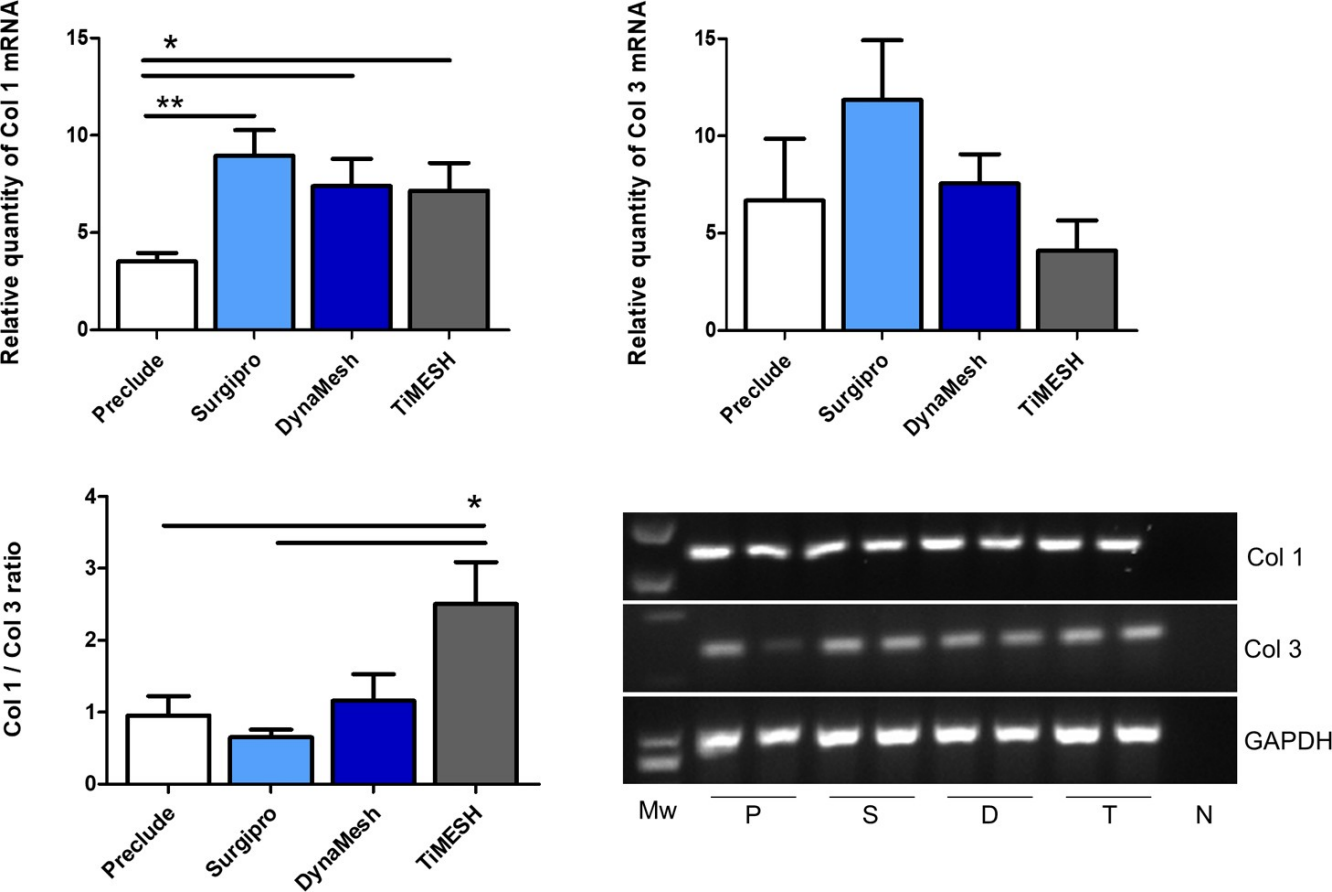
Collagen 1 and 3 mRNA expression levels determined by RT-PCR in non-injured omentum. Relative mRNA levels of collagen 1, collagen 3 and Col1/Col 3 ratios in samples of omentum (non-injured and not involved in adhesions) from animals implanted with different meshes. Gene expression was normalized to the expression recorded for the reference gene GAPDH. Agarose gel electrophoresis of RT-PCR products is shown: P, Preclude; S, Surgipro; D, DynaMesh; T, TiMESH. Mw: Molecular weight markers. N: Negative. All reticular meshes showed statistically significant higher collagen 1 mRNA expression levels compared to the laminar Preclude (**, p<0.01 vs. Surgipro and *, p<0.05 vs. DynaMesh, TiMESH). Col 1/ Col 3 ratios were greatest in TiMESH vs. Preclude or Surgipro (*, p<0.05).

mRNA expression levels for collagen 1 and collagen 3 in non-injured omental tissue displayed a similar trend. When comparing collagen 1 mRNA expression, transcript levels were significantly lower for Preclude with respect to the three reticular meshes (p<0.01, Preclude *vs*. Surgipro; p<0.05, Preclude *vs*. DynaMesh, TiMESH) (Fig 13). No significant differences were identified among the different groups for collagen 3 mRNA expression. The Col 1/Col 3 ratio showed the highest expression in TiMESH, which was statistically significant when compared to Preclude and Surgipro (p<0.05) (Fig 13).

## Discussion

Regarding hernia defect repair in the abdominal wall, the peritoneal interface remains of primary importance due to the contact between the prosthetic material and the cavity contents. Adhesiogenesis at this interface can trigger serious complications in the patient, such as an intestinal obstruction and, even more critically, the appearance of an intestinal fistula.

The selection of the right mesh material is essential to avoid these possible complications. The unsatisfactory behavior of polypropylene reticular meshes in contact with the peritoneal cavity has been proven in various contexts. This is due to the structure of the material and not to its inherent chemical composition [22,23]. In fact, when laminar polypropylene as part of a composite has been employed, the behavior at the peritoneal interface has been found to be optimal [9]. Despite some studies supporting the use of polypropylene meshes on the intraperitoneal side [24], the global clinical experience agrees that it is best to not use reticular polypropylene meshes for implantation in this position [25]. For this reason, alternative options, such as composite meshes, have been developed. Composites include a physical or chemical barrier to prevent the formation of adhesions when placed in contact with the bowels, thus showing an improved performance in this location [26]. More recently, so-called hybrid meshes have been released. This type of mesh, while keeping the reticular structure, is specifically designed to be placed on the intraperitoneal side.

One of these hybrid meshes is DynaMesh IPOM; apart from polypropylene, it contains a polyvinylidene fluoride polymer interwoven. Another alternative hybrid mesh is TiMESH, a polypropylene mesh with a coating of the inert element titanium over the PP filaments. Both meshes feature a reticular structure. Therefore, we evaluated their performance in an intraperitoneal context, in light of the existing controversies regarding the use of reticular meshes at this interface. In the design of our study, we used a bare polypropylene reticular mesh as well as an ePTFE laminar mesh as controls, considering that this latter shows optimal behavior at the peritoneal interface.

To assess the suitability of these meshes for intraperitoneal placement, we performed a sequential evaluation so that we could analyze the adhesions produced as well as their evolution. Additionally, we performed a detailed analysis of the particular features of all these meshes that could have consequences not only in the development of adhesions but also in the incorporation of this material into the host tissue when implanted in the peritoneal cavity.

In this sense, our first objective was the estimation of the mesothelialization capacity of the surface of these meshes. The appearance of a neoperitoneum in the peritoneal side over the mesh at early stages after the surgical implantation provides a protective cover that prevents the erosion of the intracavitary organs, and thus the adhesion formation. Therefore, the ability of a material to be covered by a mesothelial monolayer *in vitro* is an important indicator of its future performance when implanted *in vivo*. Our results showed no differences among the reticular meshes, with adequate adhesion and cellular expansion on the material filaments although a continuous mesothelial monolayer was not achieved due to the presence of pores in these structures. This fact suggests that the abdominal wall underlying the mesh is exposed and accessible through these pores and, furthermore, implies that non-covered areas in the material could damage the surface of the intracavitary organs.

These *in vitro* observations correlated with our subsequent findings *in vivo*. The evaluation of the peritoneal cavity after mesh implantation in experimental animals showed similar adhesion formation for bare PP and the two hybrid meshes analyzed, at both 7 and at 14 days post-implantation. The three reticular meshes (Surgipro, DynaMesh and TiMESH) led to similar adhesion formation not only from a quantitative point of view, regarding the percentage of the mesh covered with adhesions, but also qualitatively, with similar severities of the adhesions formed and similar organs involved. This supports the hypothesis that the structure of the material is the main feature influencing adhesion development, since the presence of a non-reactive coating or interweaving of a non-adhesiogenic component at the visceral side does not lead to reduced adhesion percentages when the reticular structure is maintained. Fortelny et al. [15] also reported high adhesion formation when patients were implanted with DynaMesh. In their work, adhesions covered practically the whole area of the mesh and were related to significant clinical complications, even requiring mesh explantation in several instances. This contrasts with the superior properties shown by PVDF alone in tissue responses [27] and with the fact that this material does not adhere to abdominal structures [28]. Other authors [16, 19, 29] have demonstrated, consistent with our results, the inefficiency of PVDF or TiMESH in adhesion prevention. Moreover, intestinal obstruction cases have previously been reported for DynaMesh [18]. We did not find any case of intestinal obstruction, possibly because of the slightly aggressive experimental model we employed, but adhesion formation was substantial for all the reticular meshes. Surprisingly, the available literature offers conflicting results showing reduced adhesion areas and scores in several cases for these hybrid meshes [30, 31].

By contrast to the reticular meshes, we found null adhesion formation for the ePTFE mesh (Preclude), consistent with the lack of pores in its structure and the laminar mesothelial layer distributed over it. This covering would confer a smooth serous membrane characterized by a slippery non-adhesive surface with low friction and the typical lubricant properties of mesothelial cells. The low adhesiogenic capacity of ePTFE is well-established, although some authors have surprisingly reported higher adhesion scores for ePTFE compared to TiMESH [14] or DynaMesh [12], and similar to that of pure PP [12].

We analyzed the surfaces of implants at the final study time point (14 days after the surgical procedure) by histological and scanning electron microscopy techniques. Areas free from adhesions in the reticular meshes, even if presenting a continuous mesothelial layer to a greater or lesser extent, displayed blood remains over it and a tissue arrangement following the outline of the filaments. This gave rise to a rough surface, in contrast to that observed for the laminar mesh.

The reasoning behind coating meshes with non-reactive materials is not only to avoid it coming into contact with other tissues, but also to diminish the inflammatory response of the organism to a foreign material, based on the idea that inert materials provoke a limited inflammatory reaction [13, 32]. Contrary to expectations, TiMESH showed the highest number of macrophages and foreign-body giant cells around its filaments in our experiments. The increased presence of giant cells has also been found by other researchers [33] when comparing TiMESH with a non-coated polypropylene mesh. In our opinion, the knitting pattern of this mesh could underlie the greatest presence of these cells in the implant area. Scanning electron microscopy characterization of meshes showed that, even if total textile porosity for TiMESH is greater than for Surgipro, the presence of numerous smaller pores in areas of intercrossed and overlapped filaments in TiMESH could be identified. This could translate into higher levels of macrophages in the proximity of filaments.

The severity and extent of the adhesion formation has been frequently related to the degree of inflammation within the peritoneal cavity [34]. Despite the idea that the greater the inflammatory reaction the more massive the formation of adhesions, the greatest presence of macrophages shown by the TiMESH group was not linked to a greater adhesion formation in our study.

Apart from the implants, the largest number of RAM-11- positive cells was also evident in adhesions from the TiMESH group. These cells were predominantly associated with milky spots that were numerous and displayed a high cellular proliferation in this group. The stage of milky spots activation, together with the macrophage presence at 14 days post-implantation in this group, could be indicative of a delay in the repair process. One plausible explanation for this could be that the titanium coating, even if not absorbable, is eliminated / degraded *in vivo* from the filaments over time. This would leave the polypropylene exposed and able to exert a response similar to that of uncoated polypropylene at an earlier stage. D´Amore et al. [19] recently demonstrated the degradation of anti-adhesive coatings in composite or combined meshes that could cause an enhancement in the inflammatory reaction; and therefore, late adhesion formation. Here, we demonstrated equivalent adhesion percentages for all the reticular meshes at the two study time points assayed; according to literature [35,36] and our previous experience, this value will not vary largely over time beyond this period. Hence, the hypothesis of a delayed macrophagic response in the TiMESH group appears more feasible in the present study. However, this should be confirmed through the evaluation of the inflammatory response at different study timepoints, and the long-term consequences should be elucidated further. In this sense, the selection of just two early study time points could be considered a potential shortcoming of our study in relation to the assessment of the inflammatory response progression. By contrast, our previous experience [37] with the experimental model performed here indicates that 7 and 14 postoperative days can be considered as adequate timepoints to evaluate adhesion formation. At these times, adhesions constitute a permanent tissue and the mesothelial layer in adhesion-free areas has been recovered, which prevents the formation of further adhesions.

Observations reported by different authors regarding the timing in the inflammatory process and adhesion formation after mesh placement can seem controversial. The disparities among the different experimental models found in literature can lead to discrepancies in the outcomes obtained. Factors such as the nature of the mesh (presence of absorbable/degradable components that would influence the inflammatory response), the surgical procedure (e.g. open/closed peritoneum, total/partial/no defect or the fixation method) and the animal species employed require careful consideration and hamper the comparison among different research studies.

In our case, a late progression of the repair process in the TiMESH group would also clarify the histological morphology of the adhesion tissue as an intermediate state between the original adipose tissue in the omentum and the fibrous connective tissue observed in adhesions belonging to the uncoated PP (Surgipro) group. As we previously reported [37], the macrophage presence in adhesion tissue was maximal at 3 days after Surgipro placement and diminished from this point onwards in a similar manner as occurs in the normal peritoneal reparative process [38]. In this publication [37], we indicated the correlation of macrophages with a highly cellular fibrotic tissue, concurrent with the activation of transforming growth factor-beta 1. The analysis of this and other factors that have been directly implied in adhesion development such as coagulation factors, interleukins or the vascular endothelial growth factor [39], could help us predict future consequences that the differences that we found at a molecular level between the hybrid meshes and bare PP could have in the performance of these meshes over time. These studies could also shed light on the reduced postoperative pain and analgesic consumption in the short term reported in clinical trials with TiMESH [40], despite the significant adhesion formation that it ultimately produces.

The omental milky spots activation triggered by the presence of a foreign material in the abdominal cavity was also observed in non-injured omental zones far away from the mesh. Scarce macrophages were noted in the external layer of undamaged omentum (mesothelium-submesothelium) or close to milky spots in all the experimental groups. Besides, samples from animals implanted with a reticular mesh showed higher expression levels of mRNA collagen 1 compared to those implanted with a laminar mesh. This underscores the apparent sensitivity of this tissue to any injury within the cavity, and would explain its tendency to form adhesions in the event it comes into contact with a harmful surface.

Integration of abdominal meshes into the abdominal wall determines the success of the implant, diminishing the probability of hernia recurrence and mesh migration. This is particularly relevant when testing new designs since some of the variations initially introduced to improve mesh performance, such as decreasing the density of the material or increasing its porosity, resulted in a trend towards increasing recurrence rates [41]. Clinical studies have shown satisfactory results for TiMESH in terms of recurrence [42].

Good incorporation within host tissues of all the reticular meshes examined in this study was observed through macroscopic visualization and confirmed by histological analyses. This is in contrast to the results reported by other authors [33] in which scarce mesh incorporation, basically restricted to the fixing suture sites, was found for TiMESH and a polypropylene mesh. The surgical procedure for the mesh implantation was similar to ours, with mesh placement against the closed peritoneum. The rationale for this difference between the two studies could lie in the additional attachment points that we used at the corners of the mesh. They would produce a close contact of the entire extent of the mesh with the peritoneum, allowing colonization of the material by the surrounding cells and favoring mesh integration. Even with additional fixation sites, the procedure we used was minimally invasive -as no defect was created- so that the observations made were related to the mesh itself and not to the aggressiveness of the surgical procedure.

As it reinforces the wall in the implant area, collagen analysis is of great importance in the evaluation of the performance of mesh in hernia repair. In our study, all the reticular meshes showed appropriate deposition of collagen I as well as collagen III, with the greater expression of the latter most likely due to the early stage of the repair process. The distribution of collagen in reticular meshes, in filling the space through the pores and enclosing the material, justified the appropriate integration of these meshes.

To obtain further insights into the differences in collagenization for the different meshes, analyses in gene expression were also performed for both types of collagen. Collagen 1 mRNA expression levels were greater for DynaMesh and TiMESH implants compared to Surgipro and Preclude, likely because of the larger pore size that the two former implants have. Collagen overexpression with larger-pore meshes has been observed by our group previously [43]. Collagen 3 showed higher gene expression for reticular meshes than for the laminar Preclude, although the difference was not statistically significant. The collagen 1/collagen 3 ratio in the implant areas did not show differences between the groups, which suggests a similar quality of collagen formation [44].

The experimental design that we present in this work proved to be reproducible and adequate for obtaining clear sequential visualization of adhesion formation in the same animal over time. However, our preclinical model is not devoid of certain limitations. The interspecies gap is an issue that always needs to be taken into consideration when translating findings to the clinical setting. In addition, the use of experimental animal models prevents the evaluation of certain parameters of interest, such as postoperative pain. Despite this, preclinical animal studies herein provide valuable information regarding adhesion formation and integration in mesh evaluation for hernia repair.

In conclusion, our findings indicate that:

(a) The polypropylene-containing hybrid meshes TiMESH and DynaMesh do not improve adhesion formation compared to the bare polypropylene mesh Surgipro.

(b) The reticular structure of the meshes, regardless of their composition (e.g., PP alone, PP + PVDF or PP + titanium), is determinant for the adhesion outcome because of the inability of mesothelial cells to form a continuous monolayer covering the entire surface of these meshes, which would prevent an injury of the opposing surfaces.

(c) Intraperitoneal implantation of the reticular meshes induces an increase in the mRNA Col 1 expression levels in the omentum, even when not involved in adhesions or directly injured.

(d) The three reticular meshes analyzed show excellent incorporation into host tissues. Hybrid meshes (e.g., DynaMesh and TiMESH) additionally provoke an increase in mRNA Col 1 expression in the implant area compared to PP alone or the laminar mesh Preclude.

## Supporting information

S1 Text Description of the Tensile strength experiment. (DOCX)

S1 Fig Tensile strength (N/cm) of the different meshes. δ: p<0.001 *vs*. Surgipro, DynaMesh; Φ: p<0.05 *vs*. TiMESH.
(TIF)

S2 Fig Load (N) / Elongation (mm) curves of the different meshes. (TIF)

S1 Protocol Mesothelial cell harvesting, culture and seeding protocol. (DOCX)

S2 Protocol Immunohistochemistry protocol. (DOCX)

S3 Protocol Real-time reverse transcription-polymerase chain reaction (qRT-PCR) protocol. (DOCX)
