# Supplementary material for: Evaluation of synthetic reticular hybrid meshes designed for intraperitoneal abdominal wall repair: Preclinical and in vitro behavior
Source: PLoS One. 2019 Feb 27;14(2):e0213005. doi: 10.1371/journal.pone.0213005 (PMC6392302; doi:10.1371/journal.pone.0213005)
Supplement: S1 Text — (DOCX) [file pone.0213005.s001.docx]

**TENSILE STRENGTH TESTING**

Six 10 x 1.5 (cm) strips of each mesh (Preclude, Surgipro, DynaMesh and TiMESH) were cut and tested using an INSTRON 3340 tensiometer equipped with pneumatic grips (Instron Corp., Norwood, MA, USA), with a static load of 500 N. For all measurements, the interclamp distance was 6 cm and test speed was 20 mm/min. The Load (N) / Elongation (mm) curves were provided (S2 Figure). The tensile strength (N/cm) (S1 Figure) was calculated to compare the different meshes. Data were represented by box plots (whiskers showing the minimum and maximum values) and subjected to one-way analysis of variance testing (ANOVA) and Tukey as *post hoc* test. All the statistical tests were performed using the program GraphPad Prism 5 computer package (GraphPad Software, Inc., La Jolla, CA, USA) for Windows. The level of significance was set at p<0.05.
