# Supplementary figures and images for: Evaluation of synthetic reticular hybrid meshes designed for intraperitoneal abdominal wall repair: Preclinical and in vitro behavior
Source: PLoS One. 2019 Feb 27;14(2):e0213005. doi: 10.1371/journal.pone.0213005 (PMC6392302; doi:10.1371/journal.pone.0213005)

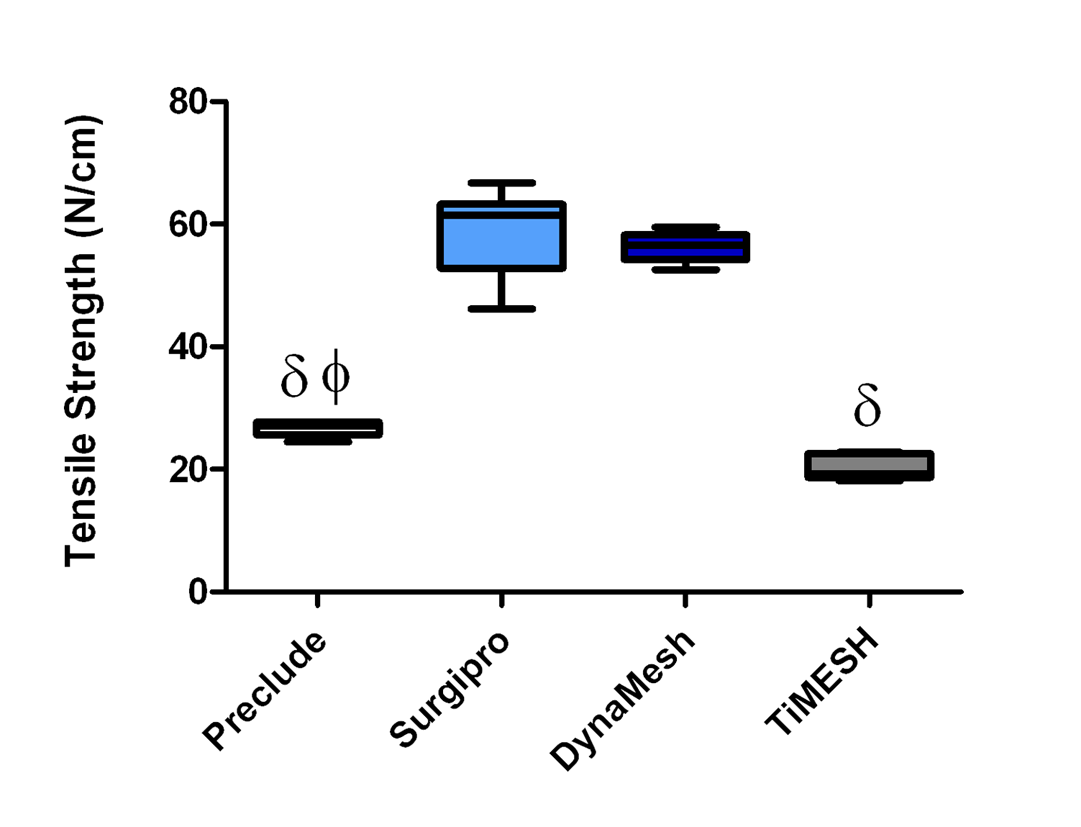

Supplement: S1 Fig — δ: p<0.001 vs. Surgipro, DynaMesh; Φ: p<0.05 vs. TiMESH. (TIF) [file pone.0213005.s002.tif]

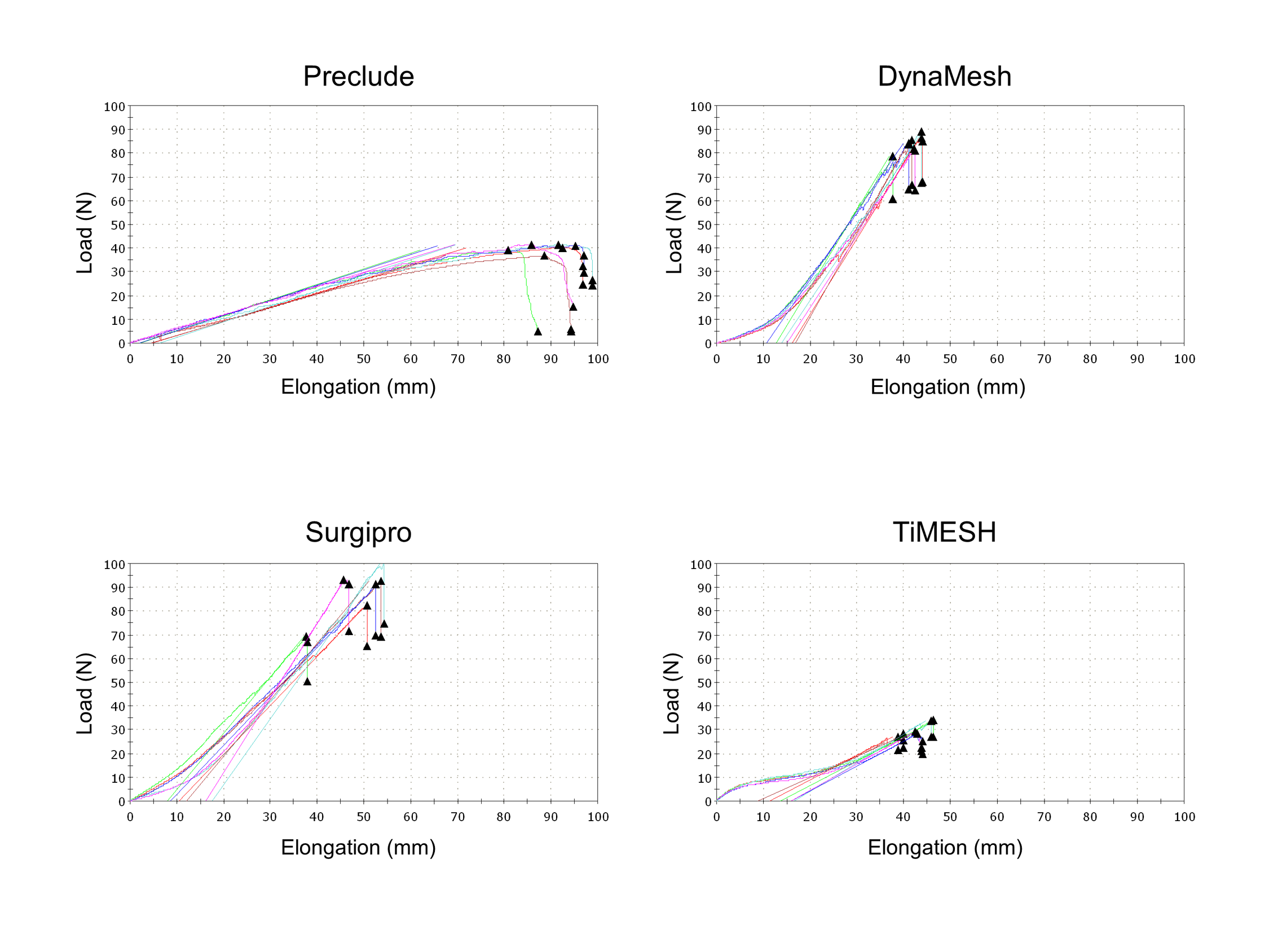

Supplement: S2 Fig — (TIF) [file pone.0213005.s003.tif]
