# Supplementary material for: Evaluation of synthetic reticular hybrid meshes designed for intraperitoneal abdominal wall repair: Preclinical and in vitro behavior
Source: PLoS One. 2019 Feb 27;14(2):e0213005. doi: 10.1371/journal.pone.0213005 (PMC6392302; doi:10.1371/journal.pone.0213005)
Supplement: S1 Protocol — (DOCX) [file pone.0213005.s004.docx]

**MESOTHELIAL CELL HARVESTING, CULTURE AND SEEDING**

Omentum was obtained from three New Zealand white rabbits that were euthanized in a CO_2_ chamber. All the protocols were developed in strict accordance with the recommendations set forth in the Guide for the Care and Use of Laboratory Animals of the National and European Institutes of Health (Spanish law 6/2013, Spanish Royal Decree 53/2013, European Directive 2010/63/UE and European Convention of the Council of Europe ETS123).

To isolate mesothelial cells (MC), fragments of omentum were washed in minimum essential medium (MEM) (Life Technologies Corporation, Carlsbad, CA, USA) to remove blood cells from the surface, and incubated in a 0.1 % collagenase type 1 solution (Life Technologies Corporation) at 37 °C for 20 min under agitation. The cell suspension was immediately centrifuged at 250x g for 10 min, the supernatant was removed, and the pelleted cells were resuspended in 10 mL of MEM and centrifuged again under the same conditions.

The cell precipitate was resuspended in 4 mL of low-glucose Dulbecco´s Modified Eagle´s Medium (DMEM) (Life Technologies Corporation) containing 10 % fetal bovine serum (FBS) (Sigma-Aldrich) and 1 % penicillin-streptomycin solution (Life Technologies Corporation). The MC were transferred to 25 cm^2^ culture flasks and incubated under controlled conditions (37 °C, 5 % CO_2_, humid atmosphere). Medium was changed every 3 days, and semiconfluent cultures were passaged using 1.5 mL of 0.25 % trypsin-ethylenediaminetetraacetic acid (EDTA) (Life Technologies Corporation) at 37 °C for 5 min. Detached MC were centrifuged at 200x g for 7 min and transferred to culture flasks at a 1:4 ratio. Cultures were visualized with a Zeiss Axiovert 40C phase-contrast microscope (Carl Zeiss, Oberkochen, Germany).

Third-passage MC were used for the mesh seeding assays. Confluent cultures were trypsinized using 1.5 mL of 0.25 % trypsin-ethylenediaminetetraacetic acid (EDTA) (Life Technologies Corporation) at 37 °C for 5 min and centrifuged at 200x g for 7 min. Pelleted cells were resuspended in 1 mL of complete DMEM, counted in a Neubauer hemocytometer via light microscopy and transferred to fibronectin-treated culture chambers (Fig 2B) at a density of 2-2.5 x 10^5^ cells/well. Complete DMEM was added to each chamber to reach a final volume of 1 mL per well. The seeded meshes were incubated under controlled conditions (37 °C, 5 % CO2, humid atmosphere), and the MC adhesion was monitored using a phase-contrast microscope (Carl Zeiss, Oberkochen, Germany).
