# Supplementary material for: Evaluation of synthetic reticular hybrid meshes designed for intraperitoneal abdominal wall repair: Preclinical and in vitro behavior
Source: PLoS One. 2019 Feb 27;14(2):e0213005. doi: 10.1371/journal.pone.0213005 (PMC6392302; doi:10.1371/journal.pone.0213005)
Supplement: S2 Protocol — (DOCX) [file pone.0213005.s005.docx]

**IMMUNOHISTOCHEMICAL ANALYSIS**

Specimens subjected to immunohistochemical analysis were fixed in F13 solution, embedded in paraffin and cut into 5-µm-thick sections. Paraffin-embedded sections were de-paraffinized in xylene, hydrated with decreasing ethanol concentrations (100%, 96% and 70%) and distilled water (for 5 min each) and equilibrated in phosphate-buffered saline (PBS; pH 7.4). Non-specific protein interactions were blocked using 3% bovine serum albumin (BSA) in PBS for 30 min at room temperature and samples were incubated with mouse monoclonal antibodies against collagen I COL-1 (ab6308; Abcam, Cambridge, UK) (1:100), collagen III hCL(III) clone III-53 (AF 5850; Medicorp, Montreal, Canada) (1:500) and the mouse monoclonal antibody against rabbit macrophages RAM-11 (M-633; Dako, Glostrup, Denmark) (1:50). Prior to incubation with the primary antibody anti-collagen I, specimens were subjected to citrate buffer antigen-retrieval for 30 minutes. The antigen-antibody reaction was detected by the alkaline phosphatase-labeled avidin-biotin procedure. This method consisted of the following steps: i) incubation with primary antibody diluted in PBS with 3% BSA overnight at 4ºC; ii) incubation with biotin-conjugated anti-mouse secondary antibody (1:300) in PBS for 90 min at room temperature; and iii) labelling with streptavidin alkaline phosphatase (1:200) in PBS for 90 min at room temperature. Negative controls were incubated with 3% BSA instead of the primary antibodies. The images were developed with the use of a chromogenic substrate containing naphthol phosphate and fast red. Cell nuclei were counterstained for 5 minutes in Carazzi´s hematoxylin. Tissue sections were examined under a light microscope (Carl Zeiss, Oberkochen, Germany).

The number of RAM-11- positive cells was quantified on implant tissue sections, considering the neoperitoneum around the biomaterial (parietal side and neotissue over the mesh towards the internal abdominal cavity). Quantification of RAM-11 positive cells was performed in 25 light microscopy fields (200x magnification) per sample in all groups by two independent observers in a blinded fashion.
