# Supplementary material for: Evaluation of synthetic reticular hybrid meshes designed for intraperitoneal abdominal wall repair: Preclinical and in vitro behavior
Source: PLoS One. 2019 Feb 27;14(2):e0213005. doi: 10.1371/journal.pone.0213005 (PMC6392302; doi:10.1371/journal.pone.0213005)
Supplement: S3 Protocol — (DOCX) [file pone.0213005.s006.docx]

**REAL-TIME REVERSE TRANSCRIPTION-POLYMERASE CHAIN REACTION (qRT-PCR)**

Fragments of the implants collected for qRT-PCR analysis included newly formed tissue from the mesh/recipient tissue interface and neoperitoneum over the mesh towards the peritoneal cavity side.

RNA was extracted through guanidine-phenol-chloroform isothiocyanate extraction procedures with TRIzol (Invitrogen, Carlsbad, CA, USA) and recovered from the aqueous phase. RNA was then precipitated with isopropanol, incubated overnight at -20°C and washed several times by centrifugation with 70% ethanol. Amounts and purity of RNA were measured at an optical density of 260/280 nm and 260/230 nm in a NanoDrop ND-1000 spectrophotometer (Thermo Fisher Scientific Inc., DE, USA) while RNA integrity was checked using 1% (wt/vol) agarose gel electrophoresis.

Complementary DNA was synthesized from 200 ng of total RNA by reverse transcription (RT) using oligo dT primers (Amersham, Fairfield, USA) and the M-MLV reverse transcriptase enzyme (Invitrogen). In parallel, a RT reaction was run without M-MLV to verify that the RNA sample lacked genomic DNA.

Complementary DNAs (cDNA) were amplified using the following primers: collagen 1A2 (col 1) (sense 5´-ATG GTG GCA CCC AGT TTG AA -3´ and antisense 5´-AGG TGA TGT TCT GAG AGG CG -3´), collagen 3A1 (col 3) (sense 5´-TGC TAA GGG TGA AGT TGG AC -3´ and antisense 5´-CCG CCA GGA CTA CCA TTG TT -3´) and GAPDH (sense 5´-TCA CCA TCT TCC AGG AGC GA-3´ and antisense 5´-CAC AAT GCC GAA GTG GTC GT-3´).

The RT-PCR mixture contained 5 µl of the inverse transcription product (cDNA) diluted 1:20, 10 µl of iQ SYBR Green Supermix (Bio-Rad Laboratories, Hercules, CA, USA), 1 µl (6 µM) of each primer (sense and antisense) and 3 µl of RNase-free water for a final reaction volume of 20 µl. RT-PCR was performed in a StepOnePlus Real-Time PCR System (Applied Biosystems, Foster City, California, USA). The samples were analyzed in triplicate. The conditions for cDNA amplification consisted of an initial stage of 10 minutes at 95°C, 40 cycles of heat denaturation at 95°C for 15 s, annealing of the primers at 60°C (collagen 1 and 3) or 55°C (GAPDH) for 30 s, and extension at 72°C for 1 minute. UltraPureTM distilled water (DNase- and RNase-free) (Invitrogen) was used as a negative control in each reaction. The products were subjected to 2% agarose gel electrophoresis, stained with a SYBR Green II RNA gel stain (Invitrogen) and visualized with UV light to confirm specificity of the primers. Gene expression was normalized against the expression value recorded for the constitutive gene glyceraldehyde 3-phosphate dehydrogenase (GAPDH).
